# Multiple Sklerose Therapie Konsensus Gruppe (MSTKG): Positionspapier zur verlaufsmodifizierenden Therapie der Multiplen Sklerose 2021 (White Paper)

**DOI:** 10.1007/s00115-021-01157-2

**Published:** 2021-07-23

**Authors:** Heinz Wiendl, Ralf Gold, Thomas Berger, Tobias Derfuss, Ralf Linker, Mathias Mäurer, Martin Stangel, Orhan Aktas, Karl Baum, Martin Berghoff, Stefan Bittner, Andrew Chan, Adam Czaplinski, Florian Deisenhammer, Franziska Di Pauli, Renaud Du Pasquier, Christian Enzinger, Elisabeth Fertl, Achim Gass, Klaus Gehring, Claudio Gobbi, Norbert Goebels, Michael Guger, Aiden Haghikia, Hans‑Peter Hartung, Fedor Heidenreich, Olaf Hoffmann, Zoë R. Hunter, Boris Kallmann, Christoph Kleinschnitz, Luisa Klotz, Verena Leussink, Fritz Leutmezer, Volker Limmroth, Jan D. Lünemann, Andreas Lutterotti, Sven G. Meuth, Uta Meyding-Lamadé, Michael Platten, Peter Rieckmann, Stephan Schmidt, Hayrettin Tumani, Martin S. Weber, Frank Weber, Uwe K. Zettl, Tjalf Ziemssen, Frauke Zipp, die Multiple Sklerose Therapie Konsensus Gruppe (MSTKG)

**Affiliations:** 1 Klinik für Neurologie mit Institut für Translationale Neurologie, Universitätsklinikum Münster, Westfälische Wilhelms-Universität Münster, Albert-Schweitzer-Campus 1, Gebäude A1, 48149 Münster, Deutschland; 2 Steuerungsgruppe der MSTKG, Münster, Deutschland; 3 Multiple Sklerose Therapie Konsensus Gruppe (MSTKG), Münster, Deutschland; 4 Neurologie, St. Josef-Hospital, Klinikum der Ruhr-Universität Bochum, Gudrunstraße 56, 44791 Bochum, Deutschland; 5 Universitätsklinik für Neurologie, Medizinische Universität Wien, Wien, Österreich; 6 Neurologische Klinik und Poliklinik, Universitätsspital Basel, Basel, Schweiz; 7 Klinik und Poliklinik für Neurologie, Universitätsklinikum Regensburg, Regensburg, Deutschland; 8 Neurologie und Neurologische Frührehabilitation, Klinikum Würzburg Mitte gGmbH, Standort Juliusspital, Würzburg, Deutschland; 9 Klinische Neuroimmunologie und Neurochemie, Klinik für Neurologie, Medizinische Hochschule Hannover, Hannover, Deutschland; 10 Klinik für Neurologie mit Institut für Translationale Neurologie, Universitätsklinikum Münster, Münster, Deutschland; 11 Klinik und Poliklinik für Neurologie, Universitätsmedizin der Johannes Gutenberg-Universität Mainz, Langenbeckstraße 1, 55131 Mainz, Deutschland

**Keywords:** Autoimmunerkrankung, Immuntherapie, Frühe Therapieintervention, Behandlungsempfehlung, Leitlinie, Autoimmune-mediated disease, Immunotherapy, Early therapeutic intervention, Treatment recommendation, Guideline

## Abstract

Die Multiple Sklerose ist eine komplexe, autoimmun vermittelte Erkrankung des zentralen Nervensystems, charakterisiert durch inflammatorische Demyelinisierung sowie axonalen/neuronalen Schaden. Die Zulassung verschiedener verlaufsmodifizierender Therapien und unser verbessertes Verständnis der Krankheitsmechanismen und -entwicklung in den letzten Jahren haben die Prognose und den Verlauf der Erkrankung deutlich verändert. Diese Aktualisierung der Behandlungsempfehlung der Multiple Sklerose Therapie Konsensus Gruppe konzentriert sich auf die wichtigsten Empfehlungen für verlaufsmodifizierende Therapien der Multiplen Sklerose im Jahr 2021. Unsere Empfehlungen basieren auf aktuellen wissenschaftlichen Erkenntnissen und gelten für diejenigen Medikamente, die in weiten Teilen Europas, insbesondere in den deutschsprachigen Ländern (Deutschland, Österreich, Schweiz), zugelassen sind.

**Zusatzmaterial online:**

Die Online-Version dieses Beitrags (10.1007/s00115-021-01157-2) enthält die vollständigen Interessenerklärungen aller Autorinnen und Autoren.

## Abstract (en)

Multiple sclerosis is a complex, autoimmune-mediated disease of the central nervous system characterized by inflammatory demyelination and axonal/neuronal damage. The approval of various disease-modifying therapies and our increased understanding of disease mechanisms and evolution in recent years have significantly changed the prognosis and course of the disease. This update of the Multiple Sclerosis Therapy Consensus Group treatment recommendation focuses on the most important recommendations for disease-modifying therapies of multiple sclerosis in 2021. Our recommendations are based on current scientific evidence and apply to those medications approved in wide parts of Europe, particularly German-speaking countries (Germany, Austria, Switzerland).

## Das wichtigste auf einen Blick

Die Multiple Sklerose (MS) ist eine komplexe, höchstwahrscheinlich autoimmun vermittelte entzündlich-neurodegenerative Erkrankung des zentralen Nervensystems, charakterisiert durch inflammatorische Demyelinisierung sowie axonalen/neuronalen Schaden. In Deutschland leiden geschätzt 250.000 Menschen an einer MS. Die Zulassung verschiedener Therapien in den letzten Jahren hat den Verlauf und die Prognose der Erkrankung wesentlich verändert. Dieses MSTKG-Positionspapier von Mitgliedern des Krankheitsbezogenen Kompetenznetzes Multiple Sklerose (KKNMS), Mitgliedern des Berufsverband Deutscher Neurologen (BDN), Mitgliedern der Deutschen Gesellschaft für Neurologie (DGN) sowie Vertretern aus Österreich und der Schweiz beschreibt auf Basis verfügbarer Evidenzen den Stand zu den wichtigsten Fragen für die verlaufsmodifizierende pharmakologische Therapie von Menschen mit MS.

Obwohl die Zulassungstexte nach wie vor zwischen schubförmig-remittierender, primär sowie sekundär progredienter MS unterscheiden, ist die klinische Einteilung der MS in 1) relapsierende sowie 2) progrediente Formen, die jeweils mit und ohne Aktivität (gemessen sowohl klinisch als auch in der Magnetresonanztomographie (MRT)) verlaufen können, der klinischen Realität aber auch der Pathobiologie näher.

„Real-World“-Kohorten- und Registerstudien der letzten Jahre zeigen, dass 1) eine frühestmögliche verlaufsmodifizierende, pharmakologische Therapieintervention langfristige Vorteile erbringt und 2) eine frühe, intensive Therapie bei Patienten mit Krankheitsaktivität Vorteile gegenüber einer Eskalationsstrategie beginnend mit niedrigerem Wirksamkeitspotenzial haben kann.

Die Auswahl der optimalen verlaufsmodifizierenden Therapie auf Basis der aktuellen Kenntnis des jeweiligen Wirkmechanismus verläuft heute bei der (hoch)aktiven MS nach zwei hauptsächlichen Behandlungsweisen. Sie beruhen auf der Evaluation des Risikos des weiteren MS-Verlaufs und von Risiken vs. Wirksamkeit verlaufsmodifizierender Therapien.

1. Die erste Variante ist ein sog. Eskalationsansatz. Hier wird mit niedrigpotenteren Medikamenten mit einem bekannten und relativ sicheren Risikoprofil begonnen und bei Nachweis von Erkrankungsaktivität trotz hinreichend langer und regelmäßiger Anwendung eine Eskalation zu potenteren Medikationen durchgeführt.
2. Die alternative Vorgehensweise ist die Initiierung mit einer Medikation höherer Wirkeffizienz, z. B. Alemtuzumab, Cladribin, Natalizumab, Ocrelizumab, Ofatumumab oder S1P-Modulatoren (zugelassen Fingolimod, Ozanimod, Ponesimod), ggf. auch schon zum Zeitpunkt der Diagnose.

Daten aus Observationsstudien lassen vermuten, dass die initiale Behandlung mit einem höher wirksamen Medikament bei Patienten mit Krankheitsaktivität mit einem niedrigeren Risiko zur Konversion in eine sekundäre progrediente MS assoziiert sein kann.

Wichtig zu Beginn oder bei der Umstellung auf eine Therapie ist das kontinuierliche Monitoring von Patienten, was nach gegenwärtigem Wissensstand eine gründliche neurologische Untersuchung sowie regelmäßige MRT-Untersuchungen des Gehirns umfasst.

Übergeordnet ist es ratsam, vor Beginn einer verlaufsmodifizierenden Therapie einen entsprechenden „De-Risking-Ansatz“ mit kompletter Labor- und Impfstatuskontrolle durchzuführen (verbindlich bei verschiedenen Medikamenten, nicht obligat bei allen).

Der Umgang mit MS-Patienten bei Impfungen, speziell in der COVID-Pandemie wird ebenfalls besprochen. Hierbei gilt, dass 1) MS-Patienten nach derzeitiger Datenlage per se kein gesteigertes Risiko für eine SARS-CoV-2-Infektion bzw. einen schweren Verlauf von COVID-19 haben, jedoch ein durch MS bedingter höherer Behinderungsgrad das Risiko für einen schweren COVID-Verlauf möglicherweise erhöhen kann, 2) die Prinzipien der verlaufsmodifizierenden Therapie und ihrer Anwendung durch die Pandemie nicht grundsätzlich verändert sind und 3) MS-Patienten klar eine Impfung empfohlen wird (siehe Stellungnahme des KKNMS).

Wir verweisen zu Wirkungen und Nebenwirkungen sowie zu den notwendigen Untersuchungen vor Einleitung der Therapie, Laborkontrollen und Therapieumstellungen auf Details in den Qualitätshandbüchern des KKNMS (entsprechend den jeweiligen Zulassungsvorgaben; https://www.kompetenznetz-multiplesklerose.de/wp-content/uploads/2021/01/KKN_2004_WEB_medikamentenhandbuch.pdf) und ihren jeweiligen Aktualisierungen.

## Einleitung und Hintergrund

Die Multiple Sklerose (MS) ist eine komplexe, höchstwahrscheinlich autoimmun vermittelte entzündlich-neurodegenerative Erkrankung des zentralen Nervensystems. In Deutschland leiden geschätzt 250.000 Menschen an dieser Krankheit [87, 123]. Die Zulassung verschiedener verlaufsmodifizierender Therapien („disease modifying therapies“, DMTs) zur Behandlung unterschiedlicher Erkrankungsformen bzw. -stadien erfordert eine Aktualisierung unseres Wissenstandes über den Nutzen bzw. die Risiken dieser Therapien sowie der Referenzen dafür.

Dieses Positionspapier der MSTKG (Multiple Sklerose Therapie Konsensus Gruppe) ist mit dem Ziel verfasst, den aktuellen Stand der wichtigsten Fragen zur MS, ihrem Verlauf und der Beeinflussung bzw. des Managements unter DMTs im Jahr 2021 auf Basis der verfügbaren wissenschaftlichen Evidenzen für die Anwendung im deutschsprachigen Raum bestmöglich wiederzugeben. In den letzten Jahren ist unser Wissen über die Erkrankung, ihre sensitive und spezifische (Früh‑)Diagnose, sowie die Verlaufs- und Prognoseeinschätzung, inklusive der Messbarkeit im klinischen Alltag enorm gewachsen. Dies hat zusammen mit der Zulassung von und der Erfahrung mit verschiedenen Präparaten und Therapiekonzepten den Stellenwert pharmakologischer Interventionen im modernen MS-Management stark geprägt. Dennoch gelingt es bisher nicht, für jede Situation eine wissenschaftlich fundierte, konkrete Empfehlung auszusprechen. Zu bestimmten Fragen liegen noch keine ausreichenden Evidenzen vor, z. B. zum Einsatz einer Pharmakotherapie beim radiologisch isolierten Syndrom (RIS). Daher ist weiterhin ein abwägendes, auf die Person und die individuellen Gegebenheiten maßgeschneidertes Vorgehen anstelle kategorischer, rigider Empfehlungen notwendig (und sinnvoll).

Die hier behandelten Kernfragen beziehen sich auf Zeitpunkt und Art therapeutischer Interventionen sowie auf die Art und Vorgehensweise des klinischen Managements im Verlauf der MS bzw. unter Therapie. Die folgenden Empfehlungen inklusive der Begründungen bilden den gegenwärtigen Stand zu Management und Therapie der MS ab. Sie sollen für den Arzt eine praktische Hilfestellung geben und eine wissenschaftlich fundierte Grundlage für aktuelle Therapieentscheidungen schaffen. Dies beinhaltet Empfehlungen zu folgenden Themenbereichen:

- die frühzeitige Behandlung von Patienten mit klinisch isoliertem Syndrom (KIS),
- die Wirksamkeit verlaufsmodifizierender pharmakologischer Therapien,
- die Behandlung von Patienten mit schubförmiger sowie auch progredienter Erkrankung,
- die Überwachung des Therapieansprechens,
- Behandlungsstrategien bei unzureichendem Therapieansprechen,
- Therapieabbruch oder -wechsel,
- Langzeiteffekte verlaufsmodifizierender Immuntherapien,
- Behandlung in besonderen Situationen wie Schwangerschaft,
- Behandlungsstrategien im Kontext von Impfungen und COVID-19.

Dieses MSTKG-Positionspapier (White Paper) ist eine Initiative von Mitgliedern des Krankheitsbezogenen Kompetenznetzes Multiple Sklerose (KKNMS), Mitgliedern des Berufsverband Deutscher Neurologen (BDN), Mitgliedern der Deutschen Gesellschaft für Neurologie (DGN) sowie Vertretern aus Österreich und der Schweiz.

Es baut in wesentlichen inhaltlichen Kernaussagen auf der Leitlinie der ECTRIMS/EAN aus dem Jahr 2018 [78] bzw. deren anstehender aktualisierter Fassung auf und präzisiert bzw. aktualisiert diese für die Versorgungssituation im deutschsprachigen Raum (für die jährlich konkrete Umsetzungshandreichungen in Form aktualisierter Qualitätshandbücher des KKNMS bestehen). Eine Reihe von Autoren dieses Positionspapiers war im Jahr 2018 an der Verfassung der ECTRIMS/EAN-Guideline beteiligt. Weiterhin erfolgte eine Auseinandersetzung mit der amerikanischen Leitlinie aus dem Jahr 2018 [11, 94].

Für einen verbesserten Lesefluss wird im gesamten Text auf Genderformulierungen verzichtet; es sind immer alle Geschlechter gemeint, außer bei expliziter Formulierung.

Die erste Version dieses Positionspapiers und die Kernfragen wurden von dem Autorenkernteam entwickelt und in mehreren mündlichen sowie schriftlichen Abstimmungsrunden wurde ein Formulierungsvorschlag mit Konsensusempfehlungen erarbeitet. Danach wurde das Positionspapier in einem zusätzlichen Konsensprozess mit den weiteren Autoren weiterentwickelt und die Kernstatements konsentiert. Grundlage zur Mitarbeit war die Offenlegung aller Interessenskonflikte (einsehbar im Supplement des Manuskriptes). Es wurden die vorliegenden aktuellen Evidenzen zu den jeweiligen Kernfragen und Empfehlungen berücksichtigt. Das Vorgehen zur Einschätzung orientiert sich an folgenden Unterpunkten, die als Endpunkte- bzw. Bewertungskriterien für verlaufsmodifizierende Therapien bei der MS betrachtet werden:

- Verringerung des Risikos von Schüben bzw. eines erneuten Schubes,
- Verringerung des Risikos bei KIS, die Kriterien für die Diagnose einer MS zu erfüllen,
- Verringerung des Risikos einer Verschlechterung der Behinderung,
- Verringerung des Risikos von Magnetresonanztomographie(MRT)-Aktivität (neue/sich vergrößernde T2-Läsionen, gadoliniumanreichernde [Gd^+^-]Läsionen) und/oder Atrophie des zentralen Nervensystems (ZNS),
- Verbesserung des Gesundheitszustandes, wie er vom Patienten erlebt/berichtet wird (PROMS – „patient reported outcome measures“ inklusive „Quality of life“ (QoL)),
- Verringerung des Risikos einer kognitiven Beeinträchtigung,
- Häufigkeit/Schweregrad von Nebenwirkungen,
- Verringerung des Risikos einer sekundär progredienten MS (bei Patienten mit schubförmigem Verlauf),
- Zeitpunkt des Wechsels zwischen unterschiedlichen DMTs,
- Überwachung der Phase zwischen zwei DMTs,
- für Pulstherapien: Auftreten von Krankheitsaktivität während des 1. Jahres der Behandlung (d. h. vor Verabreichung eines weiteren Zyklus),
- für Pulstherapien: Nutzen und Zeitpunkt eines zusätzlichen Behandlungszyklus,
- Verringerung des Risikos einer Verschlechterung der Krankheitsaktivität und der Behinderung während und nach der Schwangerschaft.

Die Evidenzstärken wurden nach Beurteilung der zugrunde liegenden (Studien‑) Datenlage bewertet (Grad 1–5, analog zu dem System des Oxford Centre for Evidence-based Medicine).

Alle Empfehlungen entsprechen einem Konsens innerhalb der Autorengruppe (damit formal >95 %).

Die Empfehlungsstärken werden auf Basis ihrer Datengrundlage analog folgendermaßen gradiert (analog zum AWMF-Regelwerk):

1. Grad A: soll (starke Empfehlung),
2. Grad B: sollte (Empfehlung),
3. Grad C: kann (schwache Empfehlung),
4. Grad D: ist möglich („good clinical practice point“).

Im Kontext der Empfehlungen basieren alle Therapieentscheidungen immer auf einem Patienten-Arzt-Konsens (Stichwort „shared decision making“). Die Wahl einer höher wirksamen DMT ist in Absprache mit dem Patienten anhand folgender Faktoren zu treffen:

- individuelle Patientencharakteristika (insbesondere MS-Charakteristika, zu erwartende Adhärenz, aber auch Alter und Geschlecht, inklusive Aspekte der Familien- bzw. Lebensplanung),
- bestehende Komorbiditäten,
- Vortherapien,
- Nebenwirkungs- und Risikoprofil des Medikaments, inklusive notwendiger Maßnahmen zum Therapiemonitoring,
- Indikation und Kostenerstattung des Medikaments.

### MS-Diagnose, Verlaufsformen und Prognose

Die Diagnose der MS wird anhand der McDonald-Kriterien gestellt. Diese wurden nach Erstpublikation 2001 in den letzten Jahren mehrfach aktualisiert, mit der letzten Version aus dem Jahr 2017 [108]. Wesentliche Änderungen bzw. Aktualisierungen neben der weiterhin als Kernkriterium der MS geltenden Dissemination in Zeit und Raum („dissemination in time“ (DIT), „dissemination in space“ (DIS)) sind 1) die oligoklonalen Banden (OKB) als Zusatzkriterium für eine DIT, 2) das Werten von symptomatischen Läsionen für sowohl DIT als auch DIS, 3) die Äquivalenz von kortikalen und juxtakortikalen Läsionen [109, 123, 126]. Historisch berichtete Schübe sind kritisch zu bewerten, sofern nicht ein passendes elektrophysiologisches und/oder morphologisches Korrelat besteht (z. B. Parästhesien, paroxysmale Sehstörungen ohne visuell evozierte Potentiale (VEP), optische Kohärenztomographie (OCT), somatosensorisch evozierte Potentiale (SEP), MRT-Korrelate). Bei (differenzial-)diagnostischer Unsicherheit sollte die Diagnose postponiert und im Zeitverlauf entsprechend reevaluiert werden.

Obwohl die Zulassungstexte nach wie vor zwischen schubförmiger, schubförmig-remittierender, primär sowie sekundär progredienter MS unterscheiden, ist die klinische Einteilung der MS in 1) relapsierende (schubförmige) sowie 2) progrediente Formen, die jeweils mit und ohne Aktivität (gemessen sowohl klinisch als auch in der MRT) verlaufen können, der klinischen Realität, aber auch der Pathobiologie näher ([71]; Tab. 1).

**Table 1:** 

*Radiologisch isoliertes Syndrom – RIS*	–	
*Klinisch isoliertes Syndrom – KIS*	Monofokal Multifokal	
*McDonald-MS*	Monofokal Multifokal	
*MS, relapsierender/schubförmiger Typ – (RMS, RRMS)*	Mild/moderater **Verlauf** Aktiv/hochaktiver **Verlauf**	Mit **Aktivität **(MRT/Schübe) Ohne Aktivität Aktivität unbestimmt
		Mit **Progredienz** Ohne Progredienz Progredienz unbestimmt
		Mit** Residuen** Ohne Residuen
*MS, progredienter Typ – (PMS, PPMS, SPMS)*	Primär progredienter **Verlauf** Sekundär progredienter Verlauf Unklarer Verlauf	Mit **Aktivität **(MRT/Schübe) Ohne Schübe, **mit MRT-Aktivität** Ohne Aktivität Aktivität unbestimmt
		Mit **Progredienz** Ohne Progredienz Progredienz unbestimmt

*KIS* klinisch isoliertes Syndrom, *MRT* Magnetresonanztomographie, *MS* Multiple Sklerose, *PMS* progrediente MS, *PPMS* primär progrediente MS, *RIS* radiologisch isoliertes Syndrom, *RMS* relapsierende MS, *RRMS* schubförmig-remittierende MS („relapsing-remitting MS“), *SPMS* sekundär progrediente MS

Als **RIS** werden klinisch asymptomatische Patienten bezeichnet, bei denen noch kein klinisch manifestes entzündlich demyelinisierendes Schubereignis aufgetreten ist, deren MRT jedoch hochsuggestiv für das Vorliegen einer entzündlich demyelinisierenden Erkrankung ist, und ggf. zudem ein chronisch-entzündliches Liquorsyndrom vorliegt oder/und Auffälligkeiten in der Elektrophysiologie bestehen (bei Ausschluss anderer Differenzialdiagnosen). Zur grundsätzlichen Indikation einer Immuntherapie beim RIS liegen noch keine ausreichenden Daten vor. Allerdings kann es Fälle mit anhaltender, dokumentierter paraklinischer Krankheitsaktivität geben, bei denen dies in individueller Abwägung angezeigt sein kann (Off-label-Therapie). Gegenwärtig läuft hierzu eine Reihe von Studien, unter anderem mit Dimethylfumarat sowie Teriflunomid (https://www.clinicaltrials.gov/ct2/results?recrs=&cond=Radiologically+Isolated+Syndrome&term=&cntry=&state=&city=&dist=).

Als **KIS** wird ein monofokales oder multifokales erstes klinisches Ereignis bei einer bisher nicht an MS erkrankten Person bezeichnet, welches dadurch suggestiv für das Vorliegen einer MS ist. Dabei besteht in Abhängigkeit der klinischen und diagnostischen Befunde (monofokale vs. multifokale Präsentation, OKB^+^ vs. OKB^–^, MRT auffällig vs. unauffällig) ein mehr oder weniger hohes Risiko für den Übergang der isolierten Symptomatik in eine MS im Laufe der Zeit.

Eine **MS nach McDonald-Kriterien** [108] kann eingeschätzt werden als schubförmiger Typ oder als progredienter Typ. Sofern nur ein Schub vorliegt, und bislang keine klinische oder paraklinische zusätzliche Aktivität oder Progredienz nachgewiesen ist, aber die Kriterien für DIT und DIS erfüllt sind, spricht man nicht mehr von KIS, sondern von McDonald-MS (weil keine sichere Festlegung, ob schubförmig [**RMS**], schubförmig remittierend [**RRMS**] oder progredient [**PMS**], möglich ist).

Die MS kann als **mild/moderat** bzw. **aktiv oder hochaktiv** bewertet werden. Entscheidend für die Einschätzung sind i) die Schubfrequenz, ii) der MRT-Befund (Läsionslast, Läsionslokalisation) und iii) die Rückbildung der/s Schübe/Schubes, die Erkrankungsaktivität sowie die Erkrankungsschwere (gemessen an klinischen sowie radiologischen Parametern); zusätzlich sind das Alter des Patienten und seine Komorbiditäten in Betracht zu ziehen.

Die Bestimmung der **Aktivität** erfolgt anhand klinischer Schübe (Schweregrad der klinischen Symptomatik/Dauer/Rückbildungstendenz) und/oder MRT-Aktivität (kontrastmittelaufnehmende Läsionen; neue oder eindeutig vergrößerte T2-Läsionen).

Die Bestimmung der **Progression** erfolgt mithilfe mindestens jährlicher Untersuchungen. Bei nicht verfügbaren Untersuchungsergebnissen wird die Aktivität als „unbestimmt“ gewertet. Die Progression wird anhand der klinischen Beurteilung gemessen, die mindestens einmal jährlich erfolgt. Als standardisierte Instrumente zur Beurteilung klinischer Funktionen bei Patienten mit MS sind neben dem „Expanded Disability Status Scale“ (EDSS) beispielsweise der Multiple Sclerosis Functional Composite (MSFC), das Brief International Cognitive Assessment for MS (BICAMS) oder 6‑ bzw. 2‑Minuten-Gehtests etabliert.

Wenn keine Beurteilungen verfügbar sind, sind Aktivität und Progression „unbestimmt“. **Grundsätzlich sind die Einordnungen nicht kategorisch oder statisch und bedürfen der Überprüfung bzw. der Monitorierung.**

Bei der schubförmigen MS erfolgt häufig noch die Unterscheidung zwischen schubförmiger (RMS) und schubförmig remittierender MS (RRMS) und hier noch die Einschätzung, ob die jeweiligen Schübe mit oder ohne Residuen verlaufen.

Das Resultat von Schüben, die nicht vollständig remittieren und damit mit Residuen ablaufen, wird als RAW („relapse associated worsening“) bezeichnet. Behinderung, die schubunabhängig akquiriert wird, bezeichnet man als PIRA („progression independent of relapse activity“; [59]).

Bei der progredienten MS (PMS) lassen sich ebenfalls Aktivität und Progression bestimmen. Zudem wird zwischen primär progredienter MS (PPMS) sowie sekundär progredienter MS (SPMS) unterschieden. Sollte die Einschätzung nicht möglich sein, verbleibt die Einschätzung **PMS**, ggf. mit dem Zusatz „unklarer Typ“.

### Verlaufsmodifizierende pharmakologische Therapien

Derzeit stehen in Deutschland 17 zugelassene Medikamente zur Behandlung der MS zur Verfügung (für eine Übersicht der jeweiligen Medikamente und Mechanismen siehe auch Tab. 2 sowie [31, 32, 39, 63, 75]).

**Table 2:** 

	Substanz	Indikation	Wirkmechanismus	Risiken für das Immunsystem	Andere Risiken	Blutbildkontrollen	Andere Kontrollen
**Oral**	*Cladribin*	(Hoch)aktive RMS	Chloriertes Analogon des DNA-Bausteins Desoxyadenosin, reduziert sowohl ruhende als auch sich teilende Lymphozyten	(Erwünschte) Leukopenie/Lymphopenie, Anämie, leicht erhöhtes Infektionsrisiko	In den Zulassungsstudien höhere Erkrankungsrate an Krebserkrankungen als unter Placebo	Alle 2–3 Monate Blutbild und diff. BB, bei Lymphopenien Grad 3 oder 4 ggf. Infektionsprophylaxe erwägen. Bei Lymphozytenwerten unter 800/μl Aussetzen der Medikation im 2. Behandlungsjahr	Alle 2–3 Monate Kontrolle GOT, GPT, y‑GT, Bilirubin, CRP, Kreatinin und U‑Status; Schwangerschaftstest vor jedem Behandlungszyklus. Jährlich cMRT
	*Dimethylfumarat*	Milde/moderate RMS	Modulation der Zytokinexpression, Hemmung der lmmunzellproliferation, Nrf2-Aktivierung, evtl. Lymphozytenapoptose	Leukopenie/Lymphopenie, seltener Neutropenie, Infektionsrisiko	Flush-Symptomatik, gastrointestinale NW, Leberwerterhöhungen	Vor Therapiebeginn: BB mit diff. BB. Unter Therapie: diff. BB alle 6–8 Wochen im ersten Therapiejahr, anschließend alle 3–6 Monate. Aussetzen der Therapie bei abs. Lymphozyten <500/µl oder Leukozyten <3000/µl, CAVE bei Werten zwischen 500–800/µl	Vor Therapiebeginn: Elektrolyte; Infektionsstatus (HBV, HCV, HIV, VZV, ggf. Tbc); Schwangerschaftstest; Leber- und Nierenwerte; CRP. cMRT <3 Monate. Unter Therapie: Keine spezifischen sonstigen Kontrollen, allerdings – wie auch bei anderen Immuntherapien – regelmäßige Kontrollen von Laborwerten weiterhin empfohlen. Jährlich cMRT
	*Fingolimod*	(Hoch)aktive RMS	Funktioneller S1P-Modulator; Festhalten von Lymphozyten in lymphatischen Organen	(Erwünschte) Lymphopenie, Herpesvirusinfektionen, VZV-Reaktivierung, hämophagozytisches Syndrom, Kryptokokkenmeningitis, Makulaödem, Impfantworten geringfügig schlechter, leicht erhöhtes Risiko für PML und Basaliome	Kardiale Reizleitungsstörung bei Erstgabe; Leberwerterhöhung, Makulaödem, vereinzelt Hauttumoren, Hypertonie, Reduktion der Diffusionskapazität, Hypercholesterinämie	Vor Therapiebeginn: BB einschl. diff. BB. Unter Therapie: Diff.-BB nach 2–4 Wochen, anschließend alle 3 Monate. Aussetzen der Therapie bei abs. Lymphozyten <200/µl, erneuter Beginn ab 600/µl möglich	Vor Therapiebeginn: EKG; Infektionsstatus (HBV, HCV, HIV, Lues, VZV, ggf. Tbc); Schwangerschaftstest; Lebertransaminasen, Serumbilirubin; Nierenwerte; CRP. Augenärztliche, ggf. dermatologische, ggf. pulmonologische Untersuchung. cMRT <3 Monate. Unter Therapie: Kardiale Überwachung bei Erstgabe; Leberwertkontrollen nach 2–4 Wochen, anschließend alle 3–6 Monate. Aussetzen der Therapie bei Transaminasen >3 × ULN und Bilirubinerhöhung oder >5 × ULN mit oder ohne Bilirubinerhöhung. Ophthalmologische Untersuchung nach 3 Monaten empfohlen, dermatologische Kontrolle jährlich. Jährlich cMRT
	*Ozanimod*	(Hoch)aktive RMS	Funktioneller S1P-Modulator; Festhalten von Lymphozyten in lymphatischen Organen	Leukopenie/Lymphopenie, leicht erhöhtes Risiko für PML (auch Fälle ohne Lymphopenie); grundsätzlich vergleichbares Risikoprofil wie für Fingolimod zu erwarten	Kardiale Reizleitungsstörung bei Erstgabe; Leberwerterhöhung, Makulaödem, vereinzelt Hauttumoren, Hypertonie, Reduktion der Diffusionskapazität, Hypercholesterinämie	Vor Therapiebeginn: BB einschl. diff. BB. Unter Therapie: Diff. BB nach 2 und 4 Wochen, anschließend alle 3 Monate. Aussetzen der Therapie bei abs. Lymphozyten <200/µl, erneuter Beginn ab 600/µl	Vor Therapiebeginn: EKG; Infektionsstatus (HBV, HCV, HIV, Lues, VZV, ggf. Tbc); Schwangerschaftstest; Lebertransaminasen, Serumbilirubin; Nierenwerte; CRP. Augenärztliche, ggf. dermatologische, ggf. pulmonologische Untersuchung. cMRT <3 Monate. Unter Therapie: Kardiale Überwachung nur bei bekannten oder im EKG festgestellten Veränderungen, Leberwertkontrollen nach 2–4 Wochen, anschließend alle 3–6 Monate. Aussetzen der Therapie bei Transaminasen >3 × ULN und Bilirubinerhöhung oder >5 × ULN mit oder ohne Bilirubinerhöhung. Ophthalmologische Untersuchung nach 3 Monaten empfohlen, dermatologische Kontrolle jährlich. Jährlich cMRT
**Oral**	*Ponesimod*	Voraussichtlich: (hoch)aktive RMS, SPMS mit Schubaktivität	Funktioneller S1P-Antagonist; Festhalten von Lymphozyten in lymphatischen Organen	(Erwünschte) Lymphopenie; erhöhtes Risiko für Infektionen (obere Atemwege; Harnwege; Herpesvirusinfektionen; Kryptokokkenmeningitis; PML), kutane Malignome; grundsätzlich vergleichbares Risikoprofil wie für Fingolimod zu erwarten	Herzrhythmusstörungen v. a. bei kardialer Vorbelastung, Leberschädigung, Einschränkung der Lungenfunktion, arterieller Hypertonus, Makulaödem, posteriore reversible Enzephalopathie (PRES)	Vor Therapiebeginn: BB einschl. diff. BB. Unter Therapie: Diff. BB nach 2 und 4 Wochen, dann alle 3 Monate. Aussetzen der Therapie bei abs. Lymphozyten <200/µl, erneuter Beginn ab 600/µl	Vor Therapiebeginn: EKG, Impfstatus, Funduskopie, Lebertransaminasen, Serumbilirubin, Infektionsstatus (HBV, HCV, HIV, VZV, Tbc), CRP, Schwangerschaftstest, Urinstatus. Augenärztliche, ggf. dermatologische, ggf. pulmonologische Untersuchung. cMRT <3 Monate. Unter Therapie: klin.-neurol. Kontrollen (nach Monat 1, dann alle 3–6 Monate); Leberwertkontrollen einschl. Serumbilirubin (nach Monat 1, dann alle 3–6 Monate). Aussetzen der Therapie bei Transaminasen >3 × ULN und Bilirubinerhöhung oder >5 × ULN mit oder ohne Bilirubinerhöhung. Ophthalmologische Untersuchung nach 3 Monaten empfohlen, dermatologische Kontrolle jährlich. Jährlich cMRT
	*Siponimod*	SPMS mit Schubaktivität, SPMS ohne Schübe, aber mit MRT Aktivität	Funktioneller S1P-Modulator; Festhalten von Lymphozyten in lymphatischen Organen	(Erwünschte) Lymphopenie, Herpesvirusinfektionen, VZV-Reaktivierung, hämophagozytisches Syndrom, Kryptokokkenmeningitis, leicht erhöhtes Risiko für PML (da bei Fingolimod aufgetreten), Impfantwort geringfügig schlechter, leicht erhöhtes Basaliomrisiko zu erwarten	Kardiale Reizleitungsstörung bei Erstgabe; Leberwerterhöhung, Makulaödem, vereinzelt Hauttumoren, Hypertonie, Reduktion der Diffusionskapazität, Hypercholesterinämie	Vor Therapiebeginn: BB einschl. diff. BB. Unter Therapie: Diff. BB nach 2 und 4 Wochen, anschließend alle 3 Monate. Bei 2 mg Dosierung und bestätigt abs. Lymphozyten <200/µl: Dosisreduktion auf 1 mg; bei 1 mg Dosierung und abs. Lymphozyten <200/µl Aussetzen der Therapie, erneuter Beginn ab 600/µl	Vor Therapiebeginn: CYP2C9 Genotypisierung; EKG, Impfstatus, Funduskopie, Lebertransaminasen, Serumbilirubin, Infektionsstatus (HBV, HCV, HIV, Lues, VZV, Tbc), CRP, Schwangerschaftstest, Urinstatus. Augenärztliche, ggf. dermatologische, ggf. pulmonologische Untersuchung. cMRT <3 Monate. Unter Therapie: Kardiale Überwachung nur bei bekannten oder im EKG festgestellten Veränderungen, Leberwertkontrollen nach 2–4 Wochen, anschließend alle 3–6 Monate. Aussetzen der Therapie bei Transaminasen >3 × ULN und Bilirubinerhöhung oder >5 × ULN mit oder ohne Bilirubinerhöhung. Ophthalmologische Untersuchung nach 3 Monaten, ggf. dermatologische, ggf. pulmonologische Kontrollen
	*Teriflunomid*	Milde/moderate RMS	DHODH-Inhibition, hierdurch Hemmung der Proliferation aktivierter Lymphozyten	Lymphopenie, Neutropenie, Infektionsrisiko, Impfantwort geringfügig schlechter, sehr selten Panzytopenie/Agranulozytose	Leberwerterhöhung, Haarausdünnung, periphere Neuropathie, akutes Nierenversagen	Vor Therapiebeginn: BB einschl. diff. BB. Unter Therapie: Diff. BB alle 2 Monate im ersten halben Therapiejahr, anschließend alle 3 Monate. Aussetzen der Therapie bei abs. Lymphozyten <200/µl	Vor Therapiebeginn: Leber‑, Pankreas‑, Nierenwerte, Impfstatus, Infektionsstatus (HBV, HCV, HIV, Lues, VZV, Tbc), CRP, Schwangerschaftstest, Urinstatus, Blutdruckkontrolle. cMRT <3 Monate. Unter Therapie: Leberwertkontrollen alle 4 Wochen in den ersten 6 Monaten, danach alle 2 Monate, bei Erhöhung der Transaminasen bestätigt über 3 × ULN Absetzen der Therapie; Kontrolle Pankreasenzyme nach Klinik; halbjährlich RR-Kontrolle; ggf. pulmonologische Kontrollen. Jährlich cMRT
	*(Azathioprin)*	Milde/moderate RMS (Reservepräparat)	Purinanalogon, hemmt DNA-/RNA-Synthese, schnell teilende Zellen besonders betroffen, Immunsuppressivum	Leukopenie/Lymphopenie, selten Neutropenie/Anämie, sehr selten thrombotische Mikroangiopathie	Erhöhtes Malignomrisiko (2 × nach 5 Jahren; 4,4 × nach 10 Jahren), Leberwerterhöhung, selten Pankreatitis	Diff. BB alle 2 Wochen, im Verlauf alle 4–8 Wochen, Zielwert Lymphopenie von 600–1000/µl	Leberwerte initial alle 2 Wochen, im Verlauf alle 4–8 Wochen. Jährlich cMRT
**Injektion**	*Glatirameracetat*	Milde/moderate RMS	Th1/Th2-Shift, APZ-Modulation, BDNF-Produktion	Reduzierte CD4/CD8-Ratio im Liquor, milde Leukozytose, Linksverschiebung, PML, leicht erhöhtes Infektionsrisiko, Impfantwort geringfügig schlechter, selten Infusionsreaktion	Sofortige Postinjektionsreaktion (SPIR) bzw. Flush, Leberwerterhöhung	Vor Therapiebeginn: BB einschl. diff. BB. Unter Therapie: Diff. BB alle 3 Monate im ersten Therapiejahr	Vor Therapiebeginn: Leber‑, Nierenwerte, Impfstatus. cMRT <3 Monate. Unter Therapie: Leber- und Nierenwertkontrollen alle 3 Monate im 1. Therapiejahr. Jährlich cMRT
	*β‑Interferon*	KIS, Milde/moderate RMS, SPMS mit Schubaktivität	Hemmung T‑Zell-Aktivierung, Treg-Induktion, Inhibition der Immunzellmigration an der BHS	Lymphknotenschwellung, Leukozytose, Leukopenie, Thrombopenie	Leberwerterhöhung, lokale Reaktion Einstichstelle, Depression; sehr selten Schilddrüsenfunktionsstörung	Vor Therapiebeginn: BB einschl. diff. BB. Unter Therapie: Diff. BB einen Monat nach Therapiebeginn, anschließend vierteljährlich. Aussetzen der Therapie bei Leukozyten <3000/µl bzw. Thrombozyten <75.000/µl	Vor Therapiebeginn: Leber‑, Nierenwerte, Impfstatus. cMRT <3 Monate. Unter Therapie: Leber- und Nierenwertkontrollen einen Monat nach Therapiebeginn, anschließend vierteljährlich. Aussetzen der Therapie bei Transaminasen >5 × ULN. Jährlich cMRT
	*Ofatumumab*	(Hoch)aktive RMS, SPMS mit Schubaktivität	Monoklonaler Antikörper gegen CD20, hierdurch Depletion der mittleren B Zell-Reihe. Vorstufen von B‑Zellen, reife Plasmazellen werden nicht eliminiert	(Erwünschte) B Zell-Lymphopenie, Verminderung von IgM im Serum, T Zell-Lymphopenie; erhöhtes Risiko für Infektionen (obere Atemwege, Harnwege, Lippenherpes), Hep. B Reaktivierung, PML	Injektionsbedingte Reaktionen	Vor Therapiebeginn: BB einschl. diff. BB und Immunstatus. Unter Therapie: Diff. BB, Immunstatus (nach Monat 3, dann alle 6–12 Monate)	Vor Therapiebeginn: IgG und IgM im Serum, Infektionsstatus (HBV, HCV, HIV, Lues, VZV, Tbc), CRP, Schwangerschaftstest, Urinstatus, Impfstatus einschl. Pneumokokkenimpfung, Ausgangs-MRT des Schädels mit Kontrastmittel (nicht älter als 3 Monate). Unter Therapie: IgG im Serum sowie Leber‑, Nierenwerte alle 6 Monate. Jährlich cMRT
**Infusion**	*Alemtuzumab*	(Hoch)aktive RMS	Monoklonaler Antikörper gegen CD52, hierdurch rasche Elimination CD52 Immunzellen in der Zirkulation, „geordnete“ Repopulation und hierdurch Immunregulation	(Erwünschte) Leukopenie/Lymphopenie, Neutropenie, Infektionsrisiko	Autoimmunerkrankungen, kardiovaskul. Risiken	Vor Therapiebeginn: BB einschl. diff. BB. Unter Therapie: Diff. BB monatlich für mind. 5 Jahre. Bei Thrombozyten <30 % des Ausgangswertes oder unterhalb der unteren Normgrenze: wöchentl. Kontrollen, bei Thrombozyten <100.000 hämatologische Abklärung	Vor Therapiebeginn: Leber‑, Nierenwerte; Infektionsstatus (HBV, HCV, HIV, Lues, VZV, Tbc), CRP, Schwangerschaftstest, Urinstatus, Impfstatus, Ausgangs-MRT des Schädels mit Kontrastmittel (nicht älter als 3 Monate). Unter Therapie: Nierenparameter (Kreatinin, GFR, U‑Status und Sediment), CRP, Leberwerte monatlich für mind. 5 Jahre, TSH alle 3 Monate. HPV-Screening bei Frauen jährlich. Jährlich cMRT
	*Natalizumab*	(Hoch)aktive RMS	Monoklonaler Antikörper gegen a4b1-Integrin, hemmt Bindung von Immunzellen an Endothelzellen via VLM-VCAM	(Erwünschte) Leukopenie/Lymphopenie, Infusionsreaktion, sekundäre antikörpervermittelte Autoimmunität (Schilddrüse, ITP, Niere), Infektanfälligkeit, Impfantwort schlechter, hohes Risiko für PML	Leberwerterhöhungen	Vor Therapiebeginn: BB einschl. diff. BB. Unter Therapie: Diff. BB alle 3–6 Monate. JCV-Antikörperstatus bei neg. Patienten alle 6 Monate, ggf. JCV-Antikörper-Index und CD62L im Verlauf	Vor Therapiebeginn: Leber‑, Nierenwerte; Ausgangs-MRT des Schädels mit Kontrastmittel (nicht älter als 3 Monate), empfohlen: Infektionsstatus (HBV, HCV, HIV, Lues, VZV, Tbc), CRP, Schwangerschaftstest, Urinstatus, Impfstatus. Unter Therapie: Leberwertkontrollen nach 3 und 6 Monaten. Aussetzen der Therapie bei Transaminasen >3 × ULN. Absetzen bei Transaminasen >5 × ULN. JCV-AK-Status nach 24 Monaten. Halbjährlich cMRT
**Infusion**	*Ocrelizumab*	(Hoch)aktive RMS, SPMS mit Schubaktivität, PPMS mit klinischer/MRT-Aktivität	Monoklonaler Antikörper gegen CD20, hierdurch Depletion unreifer und reifer B‑Zellen. Frühe Vorstufen von B‑Zellen, reife Plasmazellen sowie CD20 negative B‑Zellen werden dagegen nicht eliminiert	(Erwünschte) B‑Zell-Lymphopenie, Verminderung von IgM, ggf. auch IgG im Serum, T Zell-Lymphopenie	Infektion der oberen Atemwege, Nasopharyngitis, Influenza, Herpesinfektion	Vor Therapiebeginn: BB einschl. diff. BB; empfohlen: Immunstatus. Unter Therapie: Diff. BB (alle 3 Monate), Immunstatus 3 Monate nach Erstgabe, dann empfohlen alle 6 Monate	Vor Therapiebeginn: IgG und IgM im Serum; Leber‑, Nierenwerte; Infektionsstatus (HBV, HCV, HIV, Lues, VZV, Tbc), CRP, Schwangerschaftstest, Urinstatus, Impfstatus einschl. Pneumokokkenimpfung; Ausgangs-MRT des Schädels mit Kontrastmittel (nicht älter als 3 Monate). Während und bis zu 1 h nach der Infusion: Überwachung auf Infusionsreaktion. Unter Therapie: IgG im Serum sowie Leber‑, Nierenwerte alle 6 Monate. Jährlich cMRT
	*(Mitoxantron)*	SPMS mit Schubaktivität (Reservepräparat)	Topoisomerase-II-Inhibitor, hemmt DNA-Synthese, schnell teilende Zellen besonders betroffen, Immunsuppressivum	(Erwünschte) Neutropenie, Verminderung der Lymphozyten, Immunglobulin M im Blut erniedrigt	Übelkeit, Haarausfall, Kardiotoxizität (dosisabhängig), Leukämierisiko (nicht dosisabhängig), Infertilität, Ikterus	Vor Therapiebeginn: BB einschl. diff. BB. Unter Therapie: Diff.-BB vor jeder Gabe sowie anschließend wöchentlich für 4 Wochen. Aussetzen der Therapie bei Neutropenie <1500/ml, Dosisanpassung bei Leukopenie <2000/ml oder Thrombopenie <50.000/ml bei Nadir	Vor Therapiebeginn: Leber‑, Nierenwerte; Infektionsstatus (HBV, HCV, HIV, Lues, VZV, Tbc), CRP, Schwangerschaftstest, Urinstatus, Impfstatus, Ausgangs-MRT des Schädels mit Kontrastmittel (nicht älter als 3 Monate). Unter Therapie: Leber- und Nierenwerte, CRP, U‑Status, EKG, TTE (auch bis zu 5 Jahre nach Therapieende). Jährlich cMRT

*BB* Blutbild, *BHS* Blut-Hirn-Schranke, *CRP* C‑reaktives Protein, *DHODH* Dihydroorotatdehydrogenase, *EKG* Elektrokardiogramm, *y-GT* Gamma-Glutamyltransferase, *GFR* glomeruläre Filtrationsrate, *GOT* Glutamat-Oxalacetat-Transaminase, *GPT* Glutamat-Pyruvat-Transaminase, *HBV* Hepatitis-B-Virus, *HCV* Hepatitis-C-Virus, *HIV* humanes Immundefizienz-Virus, *HPV* humanes Papillomavirus, *IgG* Immunglobulin G, *IgM* Immunglobulin M, *KIS* klinisch isoliertes Syndrom, *MRT* Magnetresonanztomographie, *MS* Multiple Sklerose, *Nrf2* „nuclear factor erythroid 2–related factor 2“, *NW* Nebenwirkungen, *PML* progressive multifokale Leukenzephalopathie, *PMS* progrediente MS, *PPMS* primär progrediente MS, *PRES* posteriores reversibles Encephalopathie-Syndrom, *RIS* radiologisch isoliertes Syndrom, *RMS* relapsierende MS, *RR* Blutdruck, *RRMS* schubförmig-remittierende MS („relapsing-remitting MS“), *SPIR* sofortige Postinjektionsreaktion, *SPMS* sekundär progrediente MS, *S1P* Sphingosin-1-Phosphat, *Tbc* Tuberkulose, *TSH* Thyroidea stimulierendes Hormon, *TTE* transösophageale/transthorakale Echokardiografie, *ULN* upper limit of normal, *U‑Status* Urinstatus, *VZV* Varizella-Zoster-Virus

Als erste Therapieform kamen in den 1990er-Jahren verschiedene injizierbare Medikamente zur Zulassung. Sie beruhten zunächst auf rekombinant hergestellten Interferon-β-Präparaten, sowie ab 2001 zusätzlich dem Polypeptid Glatirameracetat. Interferon‑β-Präparate wurden inzwischen in Richtung einer pegylierten Form mit verlängerter Halbwertszeit weiterentwickelt [64].

Zu Beginn dieses Jahrhunderts wurden mehrere Studien zu oralen Substanzen gestartet: Fingolimod, Dimethylfumarat und Teriflunomid. Erstmalig wurden bei diesen Studien anstatt Placebo auch aktive Vergleichskomparatoren wie Interferon‑β und Glatirameracetat gewählt. In den letzten Jahren wurden weitere Studien mit Klasse I Evidenz zu den neueren, selektiveren Sphingosin-1-Phosphat (S1P)-Rezeptor-Modulatoren Ozanimod, Ponesimod und Siponimod publiziert. Durch schrittweise Dosissteigerung in der 1. Woche konnte hier bei den meisten Patienten auf eine initiale Überwachung des Herzrhythmus verzichtet werden.

Überlappend zu den oralen Therapien wurden mehrere monoklonale Antikörper (mAb) für die parenterale Therapie schubförmiger MS untersucht. Als erster mAb kam 2006 Natalizumab zur Zulassung, welcher in zwei Studien die klinische Krankheitsaktivität im Vergleich zu Placebo um fast 70 %, und die kernspintomographischen Parameter um etwa 90 % reduzieren konnte [91]. Dieser Erfolg wurde durch das Auftreten der durch das John-Cunningham-Virus (JCV) verursachten progressiven multifokalen Leukenzephalopathie (PML) bei mittlerweile mehr als 800 Patienten gedämpft, sodass regelmäßige Sicherheitsuntersuchungen mittels Antikörpertiterbestimmung gegen das HPyV‑2 („human polyomavirus“, aktuelle Bezeichnung anstelle von JCV) im Serum sowie zerebrale MRT-Untersuchungen zur Früherkennung verdächtiger Läsionen empfohlen sind (z. B. https://www.ema.europa.eu/en/medicines/human/referrals/tysabri). Natalizumab liegt seit März 2021 auch in einer subkutan zu applizierenden Formulierung vor.

In den letzten Jahren haben sich Anti-CD20-Antikörper als weiteres Therapieprinzip der schubförmigen MS etabliert. Ocrelizumab, welches eine Weiterentwicklung des bei MS formal nicht zugelassenen Rituximab darstellt, ist seit 2018 zugelassen, und das subkutan zu applizierende Ofatumumab hat im März 2021 die Zulassung erhalten. Ocrelizumab wurde zudem als erstes Präparat zum Einsatz bei PPMS zugelassen. In der relativ kleinen, aber gut strukturierten Zulassungsstudie konnte bei Patienten unter 50 Jahren, und mit kurzer Krankheitsdauer, seit Beginn der progredienten MS eine signifikante Verzögerung der Behinderungsprogression, v. a. im 1. Jahr nach Beginn der Therapie erzielt werden. Hieraus resultierte eine theoretische Verschiebung der Rollstuhlpflichtigkeit um bis zu 7 Jahre [16, 124].

Ein weiterer i.v. verabreichter Antikörper, Alemtuzumab, erreichte eine hohe Wirksamkeit in den Zulassungsstudien und gilt als Prototyp der sog. Immundepletions- und -repopulationsstrategien, ein anderer Sprachgebrauch nutzt das Wort Immunrekonstitutionstherapien (IDRP oder IRT, [73]). Bei etwa 50 % der Patienten besteht nach 2 Behandlungszyklen eine langjährige Remission ohne Notwendigkeit einer weiteren Erhaltungstherapie. Allerdings ist durch eine Reihe infektiöser und autoimmuner Nebenwirkungen die Anwendung auf hochaktive Verläufe beschränkt.

Das aus der Onkologie (Haarzellleukämie) stammende Cladribin zeigte schon zu Beginn des letzten Jahrzehnts bei RMS eine Schubreduktion von über 50 % [36]. Diese sog. Pulstherapie wird in nur 2 Wochenzyklen im Jahr 1 und 2 verabreicht. Auch dieses Präparat gehört zu den IDRPs.

Die Tab. 2 bietet eine Übersicht über die **wichtigsten Fakten zu den verfügbaren Immuntherapien der MS**, adaptiert nach Klotz et al. [63] und Wiendl et al. [123] und ergänzt um neu zugelassene Wirkstoffe. Zudem stellt das **KKNMS** für die Medikamente **Qualitätshandbücher** zu Verfügung, die in jährlich aktualisierter Weise die Anwendung bei MS-Patienten praktisch beschreiben und das Monitoring sowie den Umgang in speziellen Situationen erläutern.

Für die Schweiz gelten für die individuellen Medikamente teilweise andere Zulassungs- und Erstattungsbedingungen. Diese sind in einem strukturierten Kommentar in Zusammenarbeit der Schweizerischen Multiple Sklerose Gesellschaft und der Schweizerischen Neurologischen Gesellschaft zusammengefasst [1].

**Figure 1:**
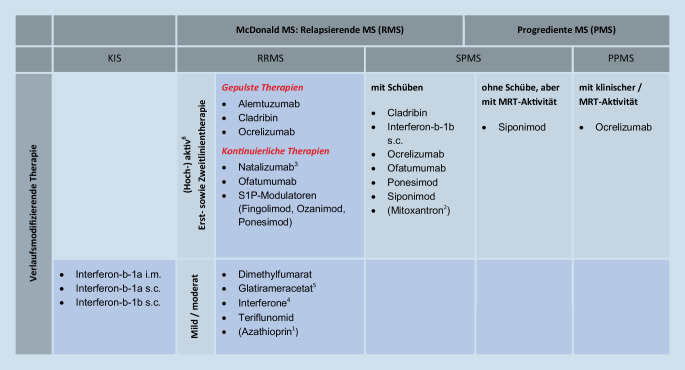

Das Therapieschema, bestehend aus allen DMTs und ihrer jeweiligen Indikation, ist in Abb. 1 skizziert. In den folgenden Abschnitten wenden wir uns den zentralen Fragen und Empfehlungen für die MS-Behandlung zu.

## Kernfragen und Empfehlungen zur therapeutischen Intervention

### (1) Welchen Nutzen hat eine DMT bei Patienten mit KIS, unabhängig davon, ob sie die Kriterien einer definitiven MS erfüllen, im Vergleich zu keiner Behandlung?

#### Review zu Evidenzen und entsprechende Empfehlungen

Als KIS wird ein monofokales oder multifokales erstes klinisches Ereignis bei einer bisher nicht an MS erkrankten Person bezeichnet, welches suggestiv für das Vorliegen einer MS ist. Typische Präsentationen sind u. a. die einseitige Optikusneuritis (ON), eine fokale Hirnstamm- oder Kleinhirnsymptomatik oder Symptome einer partiellen transversen Myelitis. Die Symptomatik entwickelt sich meist subakut und dauert mindestens 24 h an, ohne dass gleichzeitig Fieber oder eine Infektion bestehen. Sind bei der diagnostischen Abklärung die Kriterien für örtliche und zeitliche Dissemination erfüllt (was im Hinblick auf die hohe Sensitivität der im Jahr 2017 angepassten diagnostischen Kriterien [108] immer häufiger der Fall ist), markiert das KIS den mutmaßlich 1. Schub einer MS (siehe Tab. 1). Ist dies nicht der Fall, besteht in Abhängigkeit der klinischen und diagnostischen Befunde (monofokale vs. multifokale Präsentation, OKB^+^ vs. OKB^–^, MRT auffällig vs. unauffällig) ein mehr oder weniger hohes Risiko für den Übergang der isolierten Symptomatik in eine MS im Laufe der Zeit. Schwierigkeiten bestehen häufig bei Varianten der ON (immer unter der Voraussetzung, dass sich in sorgfältiger Abklärung kein anderes erklärendes Konzept findet):

- ON ohne zerebrale und/oder spinale MRT-Läsion mit negativen OKB → Diagnose isolierte ON und kein KIS,
- ON ohne zerebrale und/oder spinale MRT-Läsion mit positiven OKB → Diagnose KIS, da die OKB das Risiko für ein 2. Ereignis signifikant erhöhen,
- ON mit 1 MRT-Läsion, aber ohne OKB → Diagnose ON, da nach gegenwärtiger Definition erst ab ≥2 MRT-Läsionen die Definition eines KIS bzw. disseminierter Läsionen bestehen,
- ON mit 1 MRT-Läsion und positiven OKB → Diagnose KIS,
- ON mit ≥2 MRT-Läsionen, aber ohne positive OKB → Diagnose KIS.

Als wertvolle und aussagekräftige Quelle zur Beurteilung der weiteren Prognose von Patienten mit KIS gilt die sog. Barcelona-Kohorte, eine prospektive offene Sammlung von KIS-Patienten, die 1995 initiiert wurde. Kürzlich wurden Langzeitdaten von 401 KIS-Patienten aus dieser Kohorte publiziert [111], die vor 2006 in die Studie eingeschlossen und mindestens 10 Jahre nachbeobachtet wurden (mittlere Beobachtungszeit 14,4 Jahre). Das Risiko für eine zukünftige Behinderungsakkumulation wurde anhand der Läsionslast in der Ausgangs-MRT abgeschätzt. Insgesamt hatten Patienten mit einer frühen Behandlung (Median 4 Monate nach dem KIS) gegenüber denjenigen, die erst nach einem weiteren Schub behandelt wurden (Median 36 Monate nach dem KIS), ein signifikant geringeres Risiko, einen EDSS-Wert von 3,0 zu erreichen. Des Weiteren ergaben die Analysen, dass ca. 20 T2-Läsionen in der Baseline-MRT einen validen Prädiktor für einen aggressiven MS-Verlauf darstellen. Eine weitere in diesem Kontext wichtige Studie beschreibt den Langzeitverlauf einer englischen KIS-Kohorte über 30 Jahre, die prospektiv zwischen 1984 und 1987 rekrutiert wurde [19]. Da diese Studie häufig als Argument für eine defensivere therapeutische Herangehensweise nach erstem demyelinisierendem Ereignis herangezogen wird, ist es von Bedeutung, sich zu vergegenwärtigen, dass diese Patienten am Beginn der MRT-Ära rekrutiert wurden und die damalige Bildgebungsqualität nicht mit der heutigen Bildgebung vergleichbar ist. Dennoch konnte auch diese Arbeit eine Korrelation von Behinderungsakkumulation mit der Höhe der Läsionslast bei Baseline herstellen, insbesondere bei Vorhandensein infratentorieller MRT-Läsionen.

Welchen Nutzen eine Immuntherapie von Patienten mit KIS (unabhängig davon, ob sie die Kriterien einer definitiven MS erfüllen) hat, wurde insgesamt in 5 placebokontrollierten Studien und ihren jeweiligen Langzeitstudien untersucht. Drei Studien (mit insgesamt 1368 Patienten), die Interferonpräparate mit Placebo verglichen, zeigten für die Behandlungsgruppe eine längere Zeit bis zum nächsten Schub (=klinisch definitive MS, CDMS) und eine geringere Läsionszunahme in der MRT [25, 49, 58]. Bei den injizierbaren Therapeutika betrug die Reduktion der Schübe bei KIS ca. 40–45 %. Dies unterstützte die Bedeutung der antiinflammatorischen Aktivität insbesondere in Frühphasen der MS.

In den Langzeitstudien wurde den KIS-Patienten, die 2 Jahre Placebo erhalten hatten, Interferon angeboten. Extensionsstudien zu den β‑Interferonen deuten darauf hin, dass Patienten, die in der doppelblinden Phase ein Placebo erhielten, im Behinderungsgrad für den gesamten Beobachtungszeitraum gegenüber den Patienten, die von Anfang an mit dem Interferon‑β behandelt wurden, benachteiligt waren [58]. Die Gruppe mit der frühzeitigen Behandlung benötigte zudem eine längere Zeit bis zur Konversion zur CDMS als die Gruppe mit verzögerter Behandlung bei einer Nachbeobachtungszeit von 3 Jahren [55] – dieser Unterschied blieb bei einer Nachbeobachtungszeit von 5 [56, 61], 8 [29] und 11 Jahren bestehen [54]. Analog zeigt eine Studie zu Glatirameracetat bei KIS-Patienten (*n* = 481) im Vergleich zu Placebo ebenfalls eine verzögerte Konversion zur CDMS nach 3 Jahren [26], dies gilt auch für Studien zur Behandlung von KIS-Patienten mit Teriflunomid [77] und Cladribin [67]. Aufgrund der sensitiveren Diagnosekriterien des KIS in den revidierten Fassungen der McDonald-Kriterien sank in den letzten Jahren die Häufigkeit der Diagnose eines KIS zugunsten einer RRMS, weshalb viele der neueren Wirkstoffe nicht mehr in dieser speziellen Situation untersucht werden. Jedoch zeigt sich bei den meist retrospektiven Subgruppenanalysen aller verfügbaren Substanzen durchgehend, dass Patienten umso deutlicher profitieren, je früher sie mit einer Immuntherapie behandelt werden (siehe Therapieschema, Abb. 1).

##### Empfehlung 1

- Grundsätzlich *soll* einem KIS-Patienten (unabhängig davon, ob die Kriterien der örtlichen und zeitlichen Dissemination erfüllt sind), unter Ausschluss anderer differenzialdiagnostischer Ursachen, eine Immuntherapie angeboten werden.
- Die Auswahl der Immuntherapie *sollte *sich an den prädiktiven Parametern orientieren, wobei aktuell in erster Linie i) der MRT-Befund (Anzahl sowie Lokalisation von Läsionen), aber auch ii) Ausmaß der Rückbildung des Schubes, iii) die multifokale Präsentation und iv) liquorspezifische oligoklonale Banden bzw. chronisch-entzündliche Liquorveränderungen zu nennen sind.
- Insbesondere bei KIS-Patienten mit hoher Läsionslast und/oder infratentoriellen Läsionen in der diagnostischen MRT *sollte* angesichts der mutmaßlich ungünstigen Prognose aktiv eine Immuntherapie empfohlen werden. Hierbei ist je nach individuellen Gegebenheiten auch eine hochwirksame Therapie bereits initial in Betracht zu ziehen.
- Die Behandlung des KIS *sollte* nicht unnötig verzögert werden und *sollte* sich im individuellen (hochaktiven) Fall auch nicht an eine stufenweise Eskalation anlehnen (bitte etwaige Off-label-Nutzung beachten).

### (2) Welchen Nutzen hat eine DMT bei Patienten mit schubförmiger MS (RMS) im Vergleich zu keiner Behandlung/Behandlung mit einem anderen verlaufsmodifizierenden Medikament?

#### Review zu Evidenzen und entsprechende Empfehlungen

Alle placebokontrollierten Studien zu den aktuell verfügbaren DMTs konnten eine Reduktion der Krankheitsaktivität gemessen an Erkrankungsschüben und neuen Läsionen in der MRT gegenüber ihrem Vergleichsarm aufzeigen. Ein Teil dieser Studien konnte auch eine Überlegenheit hinsichtlich einer Reduktion der Behinderungsprogression zeigen. Die Studien der Interferonpräparate erfolgten damals an Kollektiven mit deutlich höherer Aktivität, die Schubreduktion lag ungefähr bei 30 %. Die ersten Studien mit Fingolimod zeigten neben einer großen therapeutischen Breite der Wirkdosis (zwischen 0,5 und 5 mg) auch erstmalig eine Schubreduktion um etwas mehr als 50 %. Bezüglich der Schubratenreduktion lieferten die beiden Studien mit Dimethylfumarat ebenfalls überzeugende relative Reduktionswerte. Schließlich zeigte Teriflunomid v. a. bezüglich der Stabilisierung der Behinderungsprogression in 2 Studien gute Ergebnisse. Diese 3 Präparate leiteten die 2. Generation der DMTs ein, die neben der zum Teil höheren Wirkstärke auch eine verbesserte Applikationsform und Verträglichkeit bot. Im Alltag zeigten sich Sekundärfaktoren, wie die Kompatibilität mit Familienplanung bei MS-Patientinnen (Dimethylfumarat), bessere Akutverträglichkeit (Fingolimod und Teriflunomid), autoimmunologische Komorbiditäten (Teriflunomid, Dimethylfumarat) sowie Langzeiterfahrungen, als relevant für die Entscheidungsfindung.

In den randomisierten direkten bzw. indirekten Vergleichsstudien konnte für Alemtuzumab, Fingolimod, Ozanimod, Ponesimod, Natalizumab (hier als Kombinationsstudie mit Interferon‑β gegen Natalizumab alleine), Ocrelizumab und Ofatumumab eine Überlegenheit gegenüber den jeweiligen Vergleichspräparaten hinsichtlich Schubfrequenz und MRT-Parametern gezeigt werden. Der Unterschied hinsichtlich einer Reduktion der Behinderungsprogression war hingegen nicht so deutlich, wenngleich in einigen dieser Studien ersichtlich. Insgesamt zeigt sich eine starke Evidenz für eine signifikante Reduktion der Entzündungsaktivität bei allen Präparaten.

Die verschiedenen Präparate unterscheiden sich in ihrer Wirkstärke, zumeist gemessen an der relativen Reduktion der Schubaktivität/-frequenz. Zudem lassen sie sich nach ihrer Wirkungsweise in kontinuierliche Immuntherapien und gepulste Therapien unterscheiden. Kontinuierliche Therapien haben verschiedene Wirkprinzipien von der Immunmodulation bis hin zu einer Veränderung der Immunzellmigration. Gepulste Therapien (Alemtuzumab, Cladribin, Ocrelizumab) wirken über eine Depletion von Immunzellen und über einen längeren Zeitraum (Monate bis Jahre, [73]). Allen Therapien ist gemein, dass sie primär auf das periphere adaptive Immunsystem wirken und hierdurch eine Schädigung im ZNS verhindern sollen. Ein bereits entstandener ZNS-Schaden kann nur in geringem Maße von diesen Therapien beeinflusst werden (z. B. [34, 89]). Neben ihrem Einfluss auf akut-entzündliche Aktivität in Form von Erkrankungsschüben können diese Therapien aber auch einen Effekt auf eine weitere Verschlechterung der neurologischen Funktion unabhängig von Schüben haben. Diese „progression independent of relapse activity“ (PIRA) könnte sogar der hauptsächliche Treiber neurologischer Verschlechterung während der schubförmigen Phase sein (z. B. [59, 116]).

##### Empfehlung 2

- Für die Einleitung einer DMT bei der schubförmigen MS spricht das Behandlungsziel der Reduktion entzündlicher Aktivität in Form von Erkrankungsschüben und neuen Läsionen in der MRT. Übergeordneter Fokus gilt dabei dem Erhalt der sog. zerebralen Reserve. Zudem gibt es Hinweise aus verschiedenen Studien (Registerstudien, Open-label-extension-Studien), dass diese Therapien einen positiven Einfluss auf das längerfristige Behinderungsrisiko und die sekundäre Progression haben könnten.
- Grundsätzlich *soll* Patienten mit diagnostizierter MS eine Immuntherapie angeboten werden, sofern die Therapiebegleitung durch i) adäquate Infrastruktur, ii) adäquates Krankheitsassessment, iii) kontinuierliches Monitorieren der Erkrankung, aber auch der Therapie und iv) Kenntnis, Erkennen sowie Behandlung von Therapienebenwirkungen gegeben ist. Angeboten werden kann das gesamte Spektrum zugelassener Therapien für die schubförmige MS. Für die MS steht ein breites Spektrum an DMTs zur Verfügung (Alemtuzumab, Cladribin, Dimethylfumarat, Fingolimod, Glatirameracetat/Glatirameroide, Interferon-β-1a, Interferon-β-1b, pegyliertes Interferon-β-1a, Natalizumab, Ocrelizumab, Ofatumumab, Ozanimod, Ponesimod, Teriflunomid. Reservemedikamente: Azathioprin, Mitoxantron).
- Die Auswahl der optimalen verlaufsmodifizierenden Therapie auf Basis der aktuellen Kenntnis des jeweiligen Wirkmechanismus verläuft heute nach zwei hauptsächlichen Behandlungsvorgehensweisen. Sie beruhen auf der Evaluation des Risikos des weiteren MS-Verlaufs und von Risiken vs. Wirksamkeit verlaufsmodifizierender Therapien.
  1. Die erste Variante ist ein sog. Eskalationsansatz. Hier wird mit niedrigpotenteren Medikamenten mit bekanntem und relativ sicherem Risikoprofil begonnen und bei Nachweis von weiterer Erkrankungsaktivität (klinisch oder durch eine MRT) eine Eskalation zu potenteren Medikationen durchgeführt.
  2. Die alternative Vorgehensweise ist die Initiierung mit einer Medikation höherer Wirkeffizienz, z. B. Alemtuzumab, Cladribin, Natalizumab, Ocrelizumab, Ofatumumab oder S1P-Modulatoren (Fingolimod, Ozanimod, Ponesimod), ggf. schon zum Zeitpunkt der Diagnosestellung.
- Die Auswahl der Immuntherapie *soll* sich an den Parametern der MS orientieren (Prognose, Erkrankungsaktivität sowie Erkrankungsschwere), wobei aktuell in erster Linie i) die Schubfrequenz, ii) der MRT-Befund (Läsionslast, Läsionslokalisation) und iii) die Rückbildung der/s Schübe/Schubes, die Erkrankungsaktivität sowie die Erkrankungsschwere (gemessen an klinischen sowie radiologischen Parametern) zur Beurteilung herangezogen werden; zusätzlich sind das Alter des Patienten, das Geschlecht, Vorhandensein liquorspezifischer oligoklonaler Banden bzw. chronisch-entzündlicher Liquorveränderungen, seine Komorbiditäten sowie insbesondere das Sicherheitsprofil der DMT in Betracht zu ziehen.
- Als Vorschlag zur Einschätzung einer (hoch)aktiven schubförmigen MS *sollte* gelten: ≥1 Schub innerhalb der letzten 12 Monate, ≥2 Schübe in den letzten 24 Monaten ODER ≥3 neue T2-Läsionen oder ≥1 neue Gd^+^-Läsion in einer Verlaufs-MRT (auf die Kontrastmittelgabe kann bei Vorliegen rezenter und qualitativ hochwertiger Verlaufsbilder verzichtet werden) in den letzten 12 Monaten.

### (3) Welchen Nutzen hat eine DMT bei Patienten mit progredienter MS (PPMS bzw. SPMS) im Vergleich zu keiner Behandlung?

#### Review zu Evidenzen und entsprechende Empfehlungen

Die Entwicklung von DMTs für die Behandlung der progredienten MS, sei es eine PPMS oder eine SPMS, war grundsätzlich deutlich weniger erfolgreich als bei der RMS. Es ist mittlerweile allgemein akzeptiert, dass alle DMTs früh im Krankheitsverlauf eine bessere Wirkung zeigen als im späteren Verlauf. Einer der Gründe hierfür ist wahrscheinlich eine neurodegenerative Komponente in der Pathophysiologie der MS, die auf eine Immuntherapie, deren primärer Wirkort meist nicht direkt im ZNS ist, nicht (mehr) anspricht. Für die formale SPMS sind Interferon‑β-1b, Mitoxantron sowie Siponimod zugelassen. Siponimod ist bei aktiver Erkrankung, definiert über aufgesetzte Schübe oder MRT-Aktivität, zugelassen. In den Daten der Zulassungsstudie, die explizit bei SPMS-Patienten durchgeführt wurde [53], zeigt sich ein besseres Ansprechen bei jüngeren Patienten mit entzündlicher Aktivität. Bei aufgesetzten Schüben sind formal unter dem „label“ RMS auch noch weitere DMTs zugelassen, auch wenn hier keine dezidierten Studien für Patienten mit SPMS vorliegen. Da gut gezeigt ist, dass DMTs die Schubraten reduzieren, ist daher eine DMT zumindest bei SPMS-Patienten mit Schub- bzw. MRT-Aktivität als sinnvoll zu erachten, auch wenn hierzu derzeit noch keine Langzeitbeobachtungsdaten vorliegen.

Besondere Unsicherheiten ergeben sich im weiteren Verlauf, wenn behandelte Patienten mit ursprünglich aktiver SPMS keine Schübe mehr haben. Verschiedene Experten, einschließlich der Autoren der nordamerikanischen Leitlinie [94], empfehlen, die Therapie abzusetzen, wenn eine reine Progredienz vorliegt. Unklar ist jedoch, ob möglicherweise die aufgesetzten Schübe durch eine DMT trotz fehlenden Einflusses auf die Progredienz unterdrückt werden, die Patienten also weiterhin zumindest von der Schubratenreduktion durch die DMT profitieren. Sollte eine Therapie bei Patienten mit SPMS abgesetzt werden, muss engmaschig kontrolliert werden, ob die entzündliche Aktivität im Folgenden wieder zunimmt.

Für die PPMS ist derzeit ausschließlich Ocrelizumab zugelassen. Auch hier gibt es Hinweise aus der Zulassungsstudie [79] auf eine bessere Wirksamkeit bei jüngeren Patienten mit kürzerer Krankheitsdauer. Auch wenn der Effekt bei der PPMS hinsichtlich des Gesamt-EDSS vergleichsweise gering ist, so profitieren zumindest die jüngeren Patienten von einer Therapie mit Ocrelizumab, zumal es derzeit keine zugelassene Alternative gibt. Bei älteren Patienten (>55 Jahre) mit längerem (>15 Jahre) Krankheitsverlauf und einem höheren Behinderungsgrad (EDSS-Wert >6,5) liegen keine Daten aus kontrollierten Studien vor. Dennoch sollte nach der Einschätzung der Autoren hier kein therapeutischer Nihilismus betrieben werden. Insbesondere, wenn Patienten der Verlust der physischen Selbständigkeit droht, ist ein Therapieversuch gerechtfertigt. Für die Lebensqualität der Patienten kann dieser Versuch entscheidend sein.

In der Therapie der progredienten MS werden weiterhin intrathekale Steroide (antispastisch und antientzündlich) und zyklische Methylprednisolonpulstherapien (als individueller Heilversuch) verwendet – zumeist allerdings unter dem Aspekt symptomatischer Therapieoptimierung. Hierzu existiert eine Reihe klinischer Daten (z. B. [52, 98]) sowie Erfahrungen im „Real-World“-Kontext (z. B. [30, 97], aktuelle Daten des DMSG-Registers), auch wenn keine evidenzbasierten Studien vorliegen.

##### Empfehlung 3

- Patienten mit progredienter MS profitieren insbesondere in frühen Stadien der Erkrankung von einer DMT und *sollen *behandelt werden, wenn klinische und/oder bildgebende Aktivität vorliegt. Aber auch in späteren Krankheitsphasen oder nach längerer Krankheitsdauer *sollte* im individuellen Fall eine Therapie in Erwägung gezogen werden, wenn wichtige Funktionen verloren zu gehen drohen. In der Nutzen-Risiko-Bewertung ist das Alter der Patienten mit einzubeziehen.
- Vor Beginn einer Therapie *sollen* die Therapieziele besprochen und entsprechend kontinuierlich überprüft werden. Im Falle eines unveränderten Fortschreitens der Erkrankung im Vergleich zur Situation vor Therapieinitiierung, muss von einem nicht ausreichenden Ansprechen auf die Therapie ausgegangen werden. Allerdings ist es wichtig, neben den Endpunkten der MRT-Aktivität, der Schubrate und der Gesamtbehinderung auch auf Veränderungen innerhalb relevanter Funktionssysteme des EDSS zu achten (z. B. Funktion der oberen Extremitäten) sowie auf eine Verbesserung der Lebensqualität, wie sie vom Patienten empfunden wird (QoL), und eine Verringerung des Risikos einer kognitiven Beeinträchtigung.
- Nachdem bei den progredienten Formen Veränderungen häufig nur langsam vonstattengehen, aber auch Fluktuationen zum Erkrankungsbild gehören, *sollten* positive wie negative Veränderungen bestätigt werden (idealerweise nach 3 oder 6 Monaten).
- Die Entscheidung hinsichtlich einer Wirksamkeit einer Therapie bei progredienten Verläufen *sollte* idealerweise innerhalb von 2 Jahren möglich sein. Bei fehlender Wirksamkeit einer DMT *sollte* das Absetzen der Therapie mit dem Patienten besprochen werden.

### (4) Welchen Nutzen hat eine frühe Behandlung mit einer DMT vs. keine Therapie bei MS-Patienten?

#### Review zu Evidenzen und entsprechende Empfehlungen

Es besteht Einigkeit, dass die Effekte aller DMTs früh im Verlauf der MS größer sind. Neuere Registerdaten zeigen auf, dass ein späterer Beginn einer DMT zu einer stärkeren Behinderung im Langzeitverlauf führt [18, 46, 60].

Neben der Verhinderung akuter Erkrankungsschübe ist das Ziel einer prophylaktischen Therapie die Reduktion des Risikos für längerfristige neurologische Verschlechterung bzw. eine sekundäre Progression. Aufgrund des langsamen Verlaufs einer MS kann dieses Therapieziel nicht in randomisierten Studien untersucht werden. Aus in den letzten Jahren generierten Registerdaten kann aber bereits jetzt abgeleitet werden, dass DMTs in der Tat das Risiko für eine langfristige neurologische Verschlechterung reduzieren [7, 13, 42, 46, 50, 51]. Der langfristige Nutzen einer Therapie hängt zudem davon ab, wie früh eine DMT begonnen wird [3].

Aufgrund der großen Heterogenität des klinischen Verlaufes bei der MS ist der weitere individuelle Verlauf extrem schwer vorherzusagen. Obwohl der Begriff der „benignen MS“ letztlich überwiegend verlassen wurde, kann es zu Verläufen kommen, die auch ohne Therapie nach 30 Jahren zu keiner (wesentlichen) Behinderung führen. Auch wenn in Analysen 15–20 % der Patienten längerfristig keine messbare Behinderung akkumulieren, bestehen keine zuverlässigen oder anerkannten Prädiktoren für einen Verlauf ohne wesentliche Behinderung [95].

##### Empfehlung 4

- Eine DMT bei MS *soll* so früh wie möglich nach Diagnosestellung begonnen werden, damit eine weitere/zukünftige Behinderung vermieden wird. In Einzelfällen *kann* bei Patienten mit sehr geringer Läsionslast und kompletter Rückbildung einer milden klinischen Symptomatik auch ein abwartendes Vorgehen mit regelmäßigen neurologischen und bildgebenden Kontrollen in Erwägung gezogen werden.
- Die Überlegenheit einer sofortigen Therapie nach erstmaligem Ereignis (KIS) vs. einer frühzeitigen Behandlung (<3 bis 6 Monate nach dem erstmaligen Ereignis) scheint allenfalls gering.

### (5) Welchen Vorteil hat bei MS-Patienten der frühe Beginn hochwirksamer DMTs im Vergleich zum späten Beginn?

#### Review zu Evidenzen und entsprechende Empfehlungen

Die Wahl der ersten DMT bei MS-Patienten stellt eine Herausforderung dar. Die Auswahl muss individuell erfolgen und eine Reihe von Faktoren berücksichtigen: klinische Symptomatik, MRT-Aktivität, Wirksamkeit eines Therapeutikums, Nebenwirkungen dieses Therapeutikums, Handhabung, Applikationsroute sowie die Lebenssituation und Familienplanung der Patienten. Generell kann die Faustregel aufgestellt werden, dass je wirksamer eine DMT ist, das Risiko schwerer Nebenwirkungen auch steigt. Das sog. Eskalationsschema, bei dem stets mit einem weniger wirksamen Medikament die Therapie begonnen wird und bei anhaltender Krankheitsaktivität auf eine hochwirksame DMT gewechselt wird, wurde anfänglich, als nur wenige DMTs zur Verfügung standen, propagiert [80]. Mit der Verfügbarkeit mehrerer hochwirksamer DMTs, einschließlich depletierender Therapien, wurde das „Hit-hard-and-early“-Konzept mit Einsatz dieser hocheffektiven DMTs zu Beginn der Erkrankung postuliert, in Analogie z. B. zur Rheumatologie. Kontrollierte Studien, die eine Überlegenheit eines dieser Therapiekonzepte belegen könnten, sind zwar jetzt initiiert worden, allerdings werden die Ergebnisse erst in einigen Jahren vorliegen. Retrospektive Registerstudien deuten bereits heute darauf hin, dass bei Patienten mit Krankheitsaktivität der frühzeitige Einsatz der hochwirksamen im Vergleich zu den moderat wirksamen DMTs die spätere Behinderungsprogression bzw. den Übergang in eine SPMS verzögern kann [13, 46]. Der Grund könnte hier ähnlich sein, wie bei der Verzögerung der Initiierung einer Therapie zu Beginn der Erkrankung: Unter einer weniger effektiven Therapie fortbestehende klinische oder subklinische Krankheitsaktivität kann irreversible neurologische Defizite verursachen und mit einem progressiven Krankheitsverlauf assoziierte Signalwege aktivieren, die möglicherweise durch eine potentere Therapie verhindert werden können.

Hocheffektive Therapien sind nicht für jeden Patienten geeignet und setzen eine individuelle Nutzen-Risiko-Abwägung voraus. Eine besondere Stellung haben hierbei die depletierenden bzw. Immunrekonstitutionstherapien, einschließlich der autologen hämatopoetischen Stammzelltransplantation. Sie bewirken eine tiefgreifende Veränderung des Immunsystems. Dadurch zeigen sie einerseits ein höheres Risiko schwerwiegender Nebenwirkungen, insbesondere ein in den ersten Monaten nach einem Therapiepuls erhöhtes Infektionsrisiko. Andererseits finden sich zumindest bei einem Teil der Patienten Jahre über das Therapieende hinaus anhaltende therapeutische Effekte hinsichtlich der Erkrankungsstabilisierung [14, 20, 73, 85] bis hin zu einer lange anhaltenden therapiefreien Erkrankungsstabilität. Substanzspezifisch kommen Strategien zur Risikoreduktion zur Anwendung. Im Vergleich hierzu besteht bei herkömmlichen Immuntherapien die Notwendigkeit einer kontinuierlichen Therapie mit im Zeitverlauf kumulierenden Risiken, die bei der individuellen Nutzen-Risiko-Abwägung zu bedenken sind.

##### Empfehlung 5

- Für den Langzeitverlauf zeigt sich ein Vorteil des Einsatzes hochwirksamer vs. moderat wirksamer DMTs von Beginn an. Hierfür sprechen Registerdaten, wenn auch prospektive Studien fehlen. Aufgrund möglicherweise erhöhter Risiken für z. T. schwere Nebenwirkungen und in Wichtung individueller Lebensfaktoren *sollte* der Einsatz hochwirksamer DMTs zu Beginn der Erkrankung individuell und in Abstimmung mit dem Patientenwunsch entschieden werden.
- Innerhalb der hochwirksamen DMTs gibt es das Konzept einer i) dauerhaften Therapie (Wirksamkeit relativ direkt mit Applikation und mit einer Reversibilität nach Absetzen einhergehend: Natalizumab und S1P-Rezeptor-Modulatoren [sowie Ocrelizumab und Ofatumumab mit Einschränkung aufgrund des Wirkmechanismus]) vs. einer ii) gepulsten Therapie (Wirksamkeit aufgrund Immundepletion und Repopulation deutlich über die Halbwertszeit des Medikaments hinausgehend, ggf. auch dauerhafte therapiefreie Erkrankungsstabilität: Alemtuzumab und Cladribin [sowie möglicherweise Ocrelizumab, mit starker Einschränkung aufgrund des Wirkmechanismus]).

### (6) Welche Arten von Untersuchungen/Parameter sagen bei MS-Patienten unter einer DMT ein schlechtes Ansprechen auf die Behandlung voraus?

#### Review zu Evidenzen und entsprechende Empfehlungen

Unter der zeitlich suffizienten Behandlung mit einer DMT neu aufgetretene Schübe, neue Läsionen in der MRT bzw. eine bestätigte Behinderungszunahme in den letzten 12 Monaten charakterisieren einen aktiven Verlauf und ungenügendes bzw. suboptimales Therapieansprechen. Da die DMTs jedoch unterschiedlich lange benötigen, um ihre volle Wirkung zu entfalten, und auch das Auftreten potenzieller Nebenwirkungen sehr unterschiedlich ist, sind die Behandlungs- und Monitoringzeiträume sowohl in den Zulassungs- als auch in „Real-World“-Studien unterschiedlich gewählt. In einer retrospektiven Kohortenanalyse wurde die Zeit von Therapiebeginn bis zum Wirkeintritt auf die Schubratenreduktion als ungefähr 3 bis 4 Monate beschrieben (12 bis 30 Wochen, [99, 119]), was sich mit langjährigen klinischen Erfahrungen deckt. Insbesondere bei Schub- oder MRT-Aktivität innerhalb der ersten 4 bis 8 Wochen nach Therapiebeginn sollte individuell entschieden werden, ob diese bis zu einem vollen Wirkeintritt der gestarteten DMT toleriert werden kann oder aufgrund des Einflusses auf die Erkrankungsschwere im Einzelfall bereits direkt eine Umstellung auf eine höheraktive Therapie erfolgen sollte.

Zur Evaluation des Therapieansprechens bei MS-Patienten unter einer DMT sollte neben klinischen Parametern auch die MRT-Untersuchung des Gehirns herangezogen werden [35, 118, 119]. Hierbei ist auf eine standardisierte Umsetzung und eine ausreichende Qualität der Bildgebung mit Auswertung durch einen erfahrenen Untersucher zu achten. Auswertealgorithmen können bei der Analyse der Bilder ggf. hilfreich sein, sind aber noch nicht ausreichend validiert und flächendeckend in der Praxis verfügbar.

Zusätzlich sollte bei Verdacht auf ein unzureichendes Therapieansprechen sichergestellt werden, dass seitens des Patienten eine ausreichende Medikamentenadhärenz vorliegt. Weitere Details zu den einzelnen DMTs und ihrem Monitoring sind in den Qualitätshandbüchern des KKNMS beschrieben. Zur Sicherheitskontrolle sollte bei hochwirksamen DMTs jährlich eine zerebrale MRT-Untersuchung erfolgen. Zur Überwachung der Behandlungssicherheit bei Patienten mit einem hohen Risiko für das Auftreten einer Natalizumab assoziierten PML (anti-HPyV-2[JCV]-Antikörper-positiv und Behandlungsdauer ab 18 Monaten) sind häufigere MRT-Untersuchungen in 3‑ bis 6‑monatlichen Intervallen notwendig. Bei Patienten mit hohem PML-Risiko, die die DMT wechseln, sollte vor Beginn der neuen DMT eine aktuelle MRT (nicht älter als 3 Monate) vorliegen, zudem eine präklinische PML mittels PCR im Liquor ausgeschlossen sein.

Sichere Therapieversager sind nach europäischen Konsensuspublikationen solche Patienten mit ≥3 neuen T2-Läsionen und 1 Schub oder ≥2 Schüben unabhängig von der MRT-Aktivität in 6 bis 12 Monaten trotz bestehender DMT [113, 118, 119], nach einem amerikanischen Konsensus solche Patienten mit ≥1 Schub, ≥2 neuen MRT-Läsionen oder Zunahme der Behinderung über 1 Jahr [94]. Als neue MRT-Läsionen zählen auch eindeutig vergrößerte T2-Läsionen [78].

Zu empfehlen ist demnach zur Überprüfung des Therapieansprechens bei DMT-behandelten Patienten die Durchführung und Expertenbeurteilung einer standardisierten zerebralen MRT innerhalb von 6 Monaten nach Behandlungsbeginn sowie ein Vergleich zu einer MRT nach 12 Monaten, idealerweise im Vergleich zu einem Referenzbild 3 bis 6 Monate nach Behandlungsbeginn [119]. Sollte es sich um hochaktive Krankheitsverläufe handeln oder sollten neue Symptome oder schwere Nebenwirkungen auftreten, ist die Bildgebung ggf. früher zu wiederholen. Verschiedene Zentren empfehlen ein sogenanntes Re-Baselining 3 Monate nach Therapieinitiierung, unter der Vorstellung, dass relativ häufig die Therapieinitiierung nach einem Aktivitätsereignis stattfindet und sich die eigentliche Ausgangssituation besser nach 3 Monaten definieren lässt. Primärer Parameter der Auswertung ist die Anzahl neuer oder sich vergrößernder T2-Läsionen. Eine Gd-Gabe ist zur Verlaufsbeurteilung nicht zwingend notwendig. Bei Verdacht auf ein akutes Ereignis (z. B. V. a. klinisches Schubereignis) ist allerdings die Kontrastmittelgabe weiterhin zu empfehlen. Für weitere MRT-Kontrollen in standardisierten Intervallen jenseits des Jahresabstands zum Referenzbild existiert keine ausreichende Evidenz; diese Kontrollen sind somit individuell festzulegen. Vorgeschlagen ist allerdings die 1‑mal jährliche paraklinische Kontrolle zur Dokumentation des Verlaufs, zur Einschätzung von Dynamik, Aktivität bzw. Progression.

Inwiefern und bei genau welchen klinischen Entscheidungen die Bestimmung von Neurofilamentleichtketten im Serum (sNfL) unterstützend oder gar ersetzend als Surrogatmarker eingesetzt werden kann, wird sich in den kommenden Jahren herausstellen. Momentan scheinen longitudinale Messungen hilfreich zu sein, um einen stabilen von einem aktiven Verlauf auch ohne MRT unterscheiden zu können (z. B. [2, 10]). Andere Marker, wie z. B. zerebrale Atrophie (MRT), zervikale/spinale Atrophie (MRT) oder retinale Atrophie (optische Kohärenztomographie, OCT), haben bislang noch keinen Einzug in die Routine gefunden.

Zur regelhaften Anwendung einer spinalen MRT in der Verlaufsbeurteilung der MS ergibt sich bisher keine ausreichende Evidenz. Die Erstellung einer spinalen MRT mindestens bei Diagnosestellung wird breit empfohlen, um einerseits einen Baseline-Befund zu haben und andererseits das Vorliegen spinaler Läsioner als negativen Prognosefaktor in Therapieentscheidungen einbeziehen zu können. Gleiches wie für den spinalen Befund gilt mangels ausreichend standardisierter Verfahren bisher für das Heranziehen der Hirnatrophie als Verlaufsparameter [102, 119].

##### Empfehlung 6

- Ziel der MS-Therapie ist die „bestmögliche“ Krankheitskontrolle und die bestmögliche Lebensqualität des Patienten. Praktisch *soll* die Krankheitskontrolle gemessen werden anhand klinischer Parameter (v. a. Schübe, Behinderung) sowie MRT-Aktivität (sog. NEDA-Konzept, „no evidence of disease activity“). Für die Messung der Lebensqualität stehen verschiedene Messparameter (patientenbasiert, arztbasiert) zur Verfügung.
- Die Überprüfung des Therapieerfolges *sollte* bei DMT-behandelten Patienten durch klinische Beurteilung alle 3 Monate und durch Vergleich einer standardisierten zerebralen MRT innerhalb von 3 bis 6 Monaten nach Behandlungsbeginn (gewertet als sog. Re-Baseline-MRT/Vergleichs-MRT) sowie mit einer MRT 12 Monate nach Behandlungsbeginn und danach in jährlichen Abständen erfolgen. Ein Nichtansprechen auf eine Therapie *kann* im Regelfall frühestens nach 6 bis 9 Monaten eingeschätzt werden (siehe Besonderheiten gepulster Therapien).
- Bei behinderungsrelevanten Schüben, rascher Behinderungsprogression oder schweren Nebenwirkungen (Sicherheit, Verträglichkeit) *soll* die Umstellung der DMT erwogen werden (siehe KKNMS-Qualitätshandbücher zu einzelnen Medikamenten und Umstellungen).
- Der Wechsel von einer DMT für einen mild/moderaten Verlauf zu einer DMT für einen (hoch)aktiven Verlauf *sollte* bei ≥1 relevantem Schub oder ≥2 bis 3 neuen oder relevant vergrößerten, durch Experten bestätigten MRT-Läsionen oder bei einer Zunahme der Behinderung ≥0,5 bis 1 EDSS-Punkte (bestätigt nach 3 bis 6 Monaten) innerhalb von 1 Jahr (sog. „vertikaler Switch“) vorgenommen werden.
- Ein Wechsel der DMT innerhalb der DMT-Gruppen *kann* bei Nebenwirkungen (Tolerabilität, Sicherheit) oder einer sehr geringfügigen Krankheitsaktivität (sog. „horizontaler Switch“) erfolgen.

### (7) Bei MS-Patienten unter DMT und mit Parametern, die auf ein schlechtes Ansprechen auf die Behandlung hinweisen: Was ist der Nutzen eines Wechsels zu höher wirksamen DMTs (vertikaler Wechsel) im Vergleich zu DMTs mit ähnlicher Wirksamkeit (horizontaler Wechsel)?

#### Review zu Evidenzen und entsprechende Empfehlungen

Die Behandlung der MS mit einer DMT fußt auf folgendem therapeutischem Konzept:

1. Beginn einer DMT im frühen Stadium des Krankheitsverlaufs, also nach Diagnosestellung (siehe Empfehlung 4).
2. Nachdem alle DMTs primär entzündungshemmend wirken, folgt das Therapiekonzept dem pathogenetischen Konzept, dass die effektive Verhinderung entzündlicher Krankheitsaktivität (anhand klinischer und/oder MRT-Outcomekriterien) auch zur Verhinderung weiterer/zukünftiger irreversibler Krankheitsprogression bzw. individueller Behinderung beiträgt (z. B. [82, 84]).
3. Die Wirksamkeit von DMTs wird klinisch (hinsichtlich weiterer Schübe und weiterer Zunahme der Behinderung, gemessen anhand des EDSS, ggf. auch des „Fatigue Severity Scale“ (FSS) bzw. MSFC) und radiologisch (neue oder sich vergrößernde MRT-T2-Läsionen) monitoriert (NEDA Konzept, [70]). Jedes medizinische Monitoring impliziert sinnvollerweise weitere Handlungskonsequenzen.
4. Wenn eine bestehende DMT nicht den gewünschten therapeutischen Effekt aufweist, sich also explizit weitere klinische und/oder radiologische Krankheitsaktivität zeigen (siehe Empfehlung 6), dann ist das grundsätzliche Therapieziel, nämlich keine Krankheitsaktivität, verfehlt. Im Fall des Versagens der Wirksamkeit einer moderaten DMT folgt der Wechsel auf eine höher wirksame DMT logisch und kohärent den Punkten 1) bis 3).

Die in der vorhandenen Literatur verwendete Definition des Therapieversagens einer (moderat wirksamen) DMT ist nicht einheitlich. Ungeachtet dessen gibt es neben der intuitiven Sicht (=jegliches Ausmaß neuer Krankheitsaktivität trotz bestehender Therapie) auch mittlerweile Konsens zur Bewertung des definitiven Therapieversagens [94, 113, 118, 119].

Darüber hinaus haben sich schon ältere Studien mit den Auswirkungen eines unmittelbaren vs. verzögerten Wechsels von einer moderat wirksamen DMT mit Therapieversagen auf eine hochwirksame DMT beschäftigt. Alle analysierten Studien zeigten übereinstimmend einen Vorteil beim Wechsel zu Alemtuzumab, Fingolimod oder Natalizumab im Vergleich zu Glatirameracetat/Glatirameroiden oder Interferonpräparaten [9, 12, 21, 24, 33, 47, 92, 96, 107, 115].

Alle Phase-III-Zulassungsstudien höher wirksamer DMTs zeigen in den basisdemographischen Daten der eingeschlossenen Studienpopulationen, dass ein substanzieller Anteil von bis zu 75 % der Studienteilnehmer bereits eine Vorbehandlung mit einer moderat wirksamen DMT hatte [22, 23, 36, 43, 44, 91]. Wiewohl nicht bei allen Studienteilnehmern von einer ungenügenden Wirksamkeit der früheren DMT ausgegangen werden kann (weil auch beispielsweise Nebenwirkungen ein Motiv zur Änderung der Therapie/Teilnahme an einer klinischen Studie sein konnten), so ist in Zusammenschau mit den Einschlusskriterien für diese Studien, die alle eine gewisse Krankheitsaktivität (durchschnittlich 1,3 bis 1,7 Schübe) in den 12 Monaten vor Studienbeginn verlangten, von einem erheblichen Anteil an Patienten auszugehen, die trotz ihrer moderat wirksamen DMT eine weitere Krankheitsaktivität hatten.

Außerdem haben alle Phase-III-Zulassungsstudien höher wirksamer DMTs, die in ihren jeweiligen Kernstudien mit Placebo- oder aktiven Komparatorgruppen verglichen und dann in vorgeplanten Langzeitbeobachtungsstudien weitergeführt wurden, gezeigt, dass nach Wechsel von Placebo/aktivem Komparator auf die jeweils höher wirksame DMT deren Wirksamkeit vergleichbar mit jener Studienpopulation war, die von Studienbeginn an die höher wirksame DMT erhalten hat (z. B. [15, 22, 37, 44]). Was sich dabei auch in den Beobachtungsstudien einheitlich zeigte: Die in den Placebo-/aktiven Komparatorgruppen zwischenzeitlich in der Kernstudie akkumulierte Behinderung war in weiterer Folge nicht mehr reversibel, sprich die „verlorene“ neurologische Beeinträchtigung konnte nicht mehr aufgeholt werden.

Schließlich erwies sich in einer rezenten Publikation, dass die Therapie mit einem hochwirksamen DMT ein signifikant geringeres Risiko für RAW, aber auch PIRA gegenüber einer moderat wirksamen DMT hatte [59].

Wenngleich das Konzept der NEDA Relevanz in wissenschaftlichen Bewertungen, aber auch Therapiekonzepten oder Therapiezielen hat, so ist es (bislang noch) nicht als Endpunkt für z. B. Zulassungen durch die FDA(„U.S. Food and Drug Administration“)- oder EMA(„European Medicines Agency“)- oder GBA(Gemeinsamer Bundesausschus)-Verfahren akzeptiert. Gegen das NEDA-Konzept werden verschiedene Vorbehalte geäußert. Unter Studienbedingungen stellt sich die MRT im Vergleich zu den Kriterien 10-mal sensitiver dar und dominiert daher die Berechnung. Die MRT-Endpunkte sind jedoch mit Blick auf die Häufigkeit der Untersuchungen und die Standardisierung der Beurteilung nur eingeschränkt auf die Anwendungsrealität übertragbar. Es gibt daher Befürworter einer rein klinischen NEDA-Definition (sog. NEDA2), definiert durch Schübe und Behinderungsprogression [70]. Zudem wird hinterfragt, welches Ausmaß an Kontrolle von Schubaktivität, Schubschwere und Behinderungsstabilisierung realistischerweise erreicht werden kann und ob bzw. welche paraklinische Aktivität für sich allein die Änderung des Therapieregimes rechtfertigt; ferner wie eng singuläre Messpunkte mit dem zukünftigen Verlauf der Erkrankung korrelieren (siehe dazu auch Hintergrund zu Empfehlung 6). Auch wird auf das Fehlen von Parametern hingewiesen, um das individuelle Risiko für Schübe, Behinderungsprogression oder andere Erkrankungsmanifestationen (kognitive Beeinträchtigung etc.) präzise vorherzusagen.

In Übereinstimmung mit der europäischen Leitlinie (ECTRIMS/EAN 2018) und den meisten internationalen Fachgesellschaften vertreten die Verfasser dieses Positionspapiers den Standpunkt, dass genau hier eine wesentliche Errungenschaft der modernen MS-Therapie liegt: Durch den Einsatz hochwirksamer Therapien besteht nunmehr die Möglichkeit, eine langjährige Stabilisierung der Erkrankung, ggf. sogar eine Reduktion der Krankheitsschwere zu erreichen, wenn das Entzündungsgeschehen so früh und so vollständig wie möglich gestoppt wird. Hierbei sind die bekannten (und möglicherweise noch unbekannten [Langzeit-])Risiken der DMTs abzuwägen. Aktuell stehen Nutzen-Risiko-Abwägungen häufig im Vordergrund der Diskussionen. Nach Auffassung der Autoren ist jedoch die MS und deren Kontrolle als Hauptpriorität zu gewichten, und bei der Beurteilung von Therapierisiken sollten auch die Möglichkeiten der aktiven Risikoreduktion (De-Risking) und der Sicherheitsmonitorierung positiv berücksichtigt werden. Gäbe es eine hochwirksame Therapie ohne die Gefahr von Nebenwirkungen, dann gäbe es heute kaum ein Argument, diese den Patienten vorzuenthalten. Damit ist allerdings ein wichtiger Anker für die Diskussion gesetzt, nämlich der Einfluss von Sicherheitsaspekten in Diskussionen um die Therapiekonzepte.

##### Empfehlung 7

- Bei MS-Patienten unter DMT für mild/moderate Formen und mit klinischen und/oder radiologischen Anzeichen, die auf ein schlechtes Ansprechen der aktuellen Behandlung hinweisen (siehe Empfehlungen 6), *sollte* ohne Verzögerung auf eine höher wirksame DMT gewechselt werden.
- Die Wahl der höher wirksamen DMT ist in Absprache mit dem Patienten anhand folgender Faktoren zu treffen:
  - Individuelle Patientencharakteristika (insbesondere MS-Charakteristika, aber auch Alter, Geschlecht) inklusive Aspekte der Familienplanung sowie Patientenwunsch.
  - Bestehende Komorbiditäten.
  - Nebenwirkungs- und Risikoprofil der DMT inklusive notwendiger Maßnahmen zum Therapiemonitoring.
  - Indikation und Kostenerstattung des Medikaments.

### (8) Ist bei Patienten mit schubförmiger MS, die die Behandlung mit einer hochwirksamen DMT abbrechen, das Risiko für eine Rückkehr oder einen Rebound ihrer Krankheitsaktivität erhöht?

#### Review zu Evidenzen und entsprechende Empfehlungen

In der Literatur existiert eine Vielzahl von Studien und Berichten zur weiteren Entwicklung der MS und Subaspekten wie klinischer und paraklinischer Krankheitsaktivität nach Absetzen einer DMT. Dabei ist grundsätzlich zu berücksichtigen, dass der Verlauf nach Absetzen oder Umstellung einer Therapie von verschiedenen Faktoren abhängt, wie von der Erkrankungsschwere des einzelnen Patienten, der Erkrankungsdauer, Komorbiditäten und der Art der vorherigen DMT. Während gepulste Immuntherapien eher eine längerfristige Stabilisierung der Krankheit bewirken, lassen Erhaltungstherapien nach Beendigung eine raschere Rückkehr von Krankheitsaktivität erwarten. Die Therapiesequenz ist ebenfalls von wesentlicher Relevanz (exemplarisch [68, 88, 121]). Hinzu kommen noch immunpathogenetische Faktoren (Genetik, Umwelt, Lebensstil).

In den zugrunde liegenden Betrachtungen bestehen große Unterschiede in den Wash-out-Perioden, also in den Zeiten zwischen Absetzen einer Substanz und Beginn der Behandlung mit einer neuen Substanz (typischerweise von 1 bis zu 6 Monaten). Besondere Betrachtung in diesem Kontext finden die Medikamente, die die Leukozytenwanderung beeinflussen [6, 93]. Bei ihnen wird in verschiedenen Berichten neben dem zu erwartenden Wiederauftreten der Krankheitsaktivität aufgrund des Absetzens eine hierüber hinausgehende Zunahme der Krankheitsaktivität im Sinne eines Rebounds beschrieben. Rebound bedeutet in diesem Zusammenhang eine Wiederkehr der Erkrankungsaktivität auf ein Niveau, welches dasjenige vor Beginn der Therapie überschreitet. Wenn auch verschiedene Studien diesen Effekt für Fingolimod und Natalizumab beschreiben, so ist letztlich nicht generell zu erwarten, dass es nach Absetzen dieser Therapien zu einer überschießenden Erkrankungsaktivität kommt. Allerdings sollte immer eine entsprechende Folgetherapie im Anschluss an diese Therapien verabreicht werden, da das Wiederauftreten der Krankheitsaktivität so zu verhindern sein sollte [6, 93].

##### Empfehlung 8

- Das Absetzen bzw. Aussetzen einer Medikation für die Therapie der (hoch)aktiven MS, entweder auf Basis von suboptimaler Wirksamkeit oder Sicherheitsbedenken, *soll* von einem klaren Konzept einer Folgetherapie begleitet sein.
- Bei der Auswahl der Nachfolgemedikation *sollten *folgende Faktoren berücksichtigt werden:
  1. Erkrankungsaktivität (klinisch sowie magnetresonanztomographisch). Je höher die Krankheitsaktivität, desto höher die Notwendigkeit für den unmittelbaren Start einer neuen Therapie.
  2. Erkrankungsschwere.
  3. Halbwertzeit sowie biologische Aktivität der vorhergehenden Medikation (Unterscheidung zwischen sog. Erhaltungstherapien [Natalizumab, S1P-Modulatoren, z. T. Ocrelizumab] und gepulsten Therapien [Alemtuzumab, Cladribin, z. T. Ocrelizumab]). Entsprechende Empfehlungen finden sich in den Qualitätshandbüchern des KKNMS.
  4. Darüber hinaus (s. Empfehlungen zu Frage 10) ist das Risiko einer „Carry-over“-PML bestmöglich zu reduzieren. Dabei sind klinische, magnetresonanztomographische, aber auch liquordiagnostische Parameter (Nachweis von HPyV‑2 [JCV]-DNA mittels PCR) heranzuziehen, um den Status Baseline oder Status vor Umsetzung zu bestimmen.
- Die Gefahr eines Wiederauftretens der Erkrankungsaktivität oder sogar eines Rebounds (insbesondere nach Leukozytenmigrationstherapien wie Natalizumab oder S1P-Rezeptor-Modulatoren) *soll* berücksichtigt werden. Das Wiederauftreten der Erkrankungsaktivität ist bei diesen Substanzen 2 bis 6 Monate nach Absetzen zu erwarten.

### (9) Ist bei Patienten mit schubförmiger MS, die unter DMT über einen langen Zeitraum stabil bleiben, eine Weiterbehandlung vorteilhaft im Vergleich zum Absetzen des Medikaments?

#### Review zu Evidenzen und entsprechende Empfehlungen

Die wissenschaftliche Datenlage zu dieser klinisch hochrelevanten Frage ist wenig fundiert. Es liegen retrospektive Beobachtungsstudien vor, die sich überwiegend auf die älteren injizierbaren MS-Therapeutika beziehen und meist kleinere Kohorten umfassen. Eine aus der Global MS Database stammende prospektive Arbeit von Kister et al. [62] beschreibt, dass zwar die Schubrate nach Absetzen injizierbarer MS-Therapien stabil bleibt, aber die Krankheitsprogression signifikant beschleunigt ist. Dies steht im Einklang mit kleineren retrospektiven Beobachtungen, sodass bei guter Verträglichkeit und Sicherheit der Therapie die Patienten zur Fortführung motiviert werden sollten.

Besondere Betrachtung erfordern die auf einer Hemmung der Leukozytenmigration beruhenden Wirkstoffe (S1P-Rezeptor-Modulatoren Fingolimod, Ozanimod, Ponesimod, Siponimod sowie Natalizumab; [6, 93]). Hier sollte nur in Ausnahmefällen ein Absetzen ohne Konzept für eine nachfolgende Therapie erfolgen (siehe auch Problemfeld von Schwangerschaft, Stillzeit, besondere Lebensumstände wie z. B. Operationen).

Bei den IRTs (Alemtuzumab, Cladribin, mit Einschränkung Ocrelizumab) liegt der besondere Fall vor, dass die Möglichkeit einer Erkrankungsremission ohne weiterführende oder nachfolgende Therapie zum Therapiekonzept gehört (z. B. [73]). Insbesondere die Kohorten der Zulassungsstudien von Alemtuzumab (CARE MS I und II, z. B. [20, 120]) sind unter sehr kontrollierten Bedingungen nachverfolgt worden. Sie zeigen, dass Patienten in etwa 50 % der Fälle über viele Jahre ohne die Notwendigkeit einer Nachfolgetherapie stabil sein können, aber auch im Falle des Wiederauftretens von Krankheitsaktivität nochmals mit CD52-Depletion behandelt werden können (mit Aussicht auf Erfolg der Restabilisierung). Für Cladribin sind ebenfalls Daten der CLARITY-Extension verfügbar. Auch hier zeigt eine Gruppe von Patienten nach nur 2 Depletionszyklen lange andauernde Erkrankungsstabilität in Abwesenheit einer weiteren DMT (siehe z. B. [120]). Für das Absetzen B‑Zell-depletierender Medikamente existieren derzeit wenige kontrollierte Studien. Vom Wirkmechanismus ist allerdings aufgrund der B‑Zell-Dominanz des Depletionsprinzips eine Reversibilität zu erwarten, von daher auch letztlich eine Wiederkehr der Erkrankungsaktivität. Etablierte prognostische oder diagnostische Marker (wie z. B. die Dynamik von depletierten vs. repopulierenden Immunzelltypen), die die Zugehörigkeit zu einer solchen Gruppe für dauerhafte Remission anzeigen würden, existieren für die MS nicht, sodass auch unter solchen Therapien die Vorgaben für das Monitoring und ein entsprechendes Handeln im Falle des Wiederauftretens von Erkrankungsaktivität geboten sind.

Stärkere Beachtung finden inzwischen Beobachtungen zur Relevanz von Krankheitsaktivität in Abhängigkeit vom Alter, von Effekten der Therapie in Abhängigkeit vom Alter und von Phänomenen der Immunseneszenz vs. Immunkompetenz im Alter (z. B. [27, 125]). Grob lässt sich sagen, dass die Entzündungsaktivität und auch der Effekt der Immuntherapie, insbesondere die Beeinflussung der Progression, mit dem Alter abnimmt. In Wichtung der Therapieziele und des Nutzen-Risiko-Profils kommt der Erkrankungsaktivität damit, insbesondere in höherem Alter (>55), stärkere Bedeutung zu.

##### Empfehlung 9

- Bei MS-Patienten, die unter einer bestimmten DMT und mit klinischem und/oder radiologischem Monitoring stabil sind und bei denen keine Sicherheitsprobleme oder Verträglichkeitsprobleme bestehen, *soll* die Therapie weitergeführt werden.
- Das Absetzen oder Pausieren einer Therapie ist – abhängig von dem Wirkmechanismus – mit der Gefahr des Wiederauftretens von Erkrankungsaktivität und/oder -progression assoziiert (siehe auch Empfehlungen zu Frage 8).
- Das Absetzen oder Pausieren einer Therapie bei explizitem Patientenwunsch (ohne eine geplante Folgetherapie) *kann* unter klaren Maßgaben für ein klinisches wie bildgebendes Monitoring erfolgen.

### (10) Bei Patienten, bei denen ein Wechsel der DMT geplant ist: Wie sollte die empfohlene Strategie aussehen?

#### Review zu Evidenzen und entsprechende Empfehlungen

Die Heterogenität der MS stellt bei gleichzeitiger Verfügbarkeit unterschiedlicher DMTs eine Herausforderung bei der Auswahl der Therapie dar. Ziel ist es, die Therapie auszuwählen, die den individuellen Bedürfnissen des Patienten am besten entspricht, unter Berücksichtigung der Wirksamkeit, eines akzeptablen Nutzen-Risiko-Verhältnisses und der Einfachheit der Anwendung [38]. In Ermangelung prädiktiver Biomarker sind häufig schrittweise, oft zeitaufwendige Optimierungen notwendig, die ggf. mehrfache Wechsel der DMT zur Folge haben [76]. Gründe für den Wechsel einer DMT können mangelnde Wirksamkeit, unerwünschte Ereignisse (Unverträglichkeit, Sicherheit) und unzureichende Therapieadhärenz sein. Eine kürzlich publizierte multizentrische, retrospektive italienische Studie [101] analysierte Daten von 2954 Patienten mit in den Jahren von 2010 bis 2017 neu diagnostizierter RRMS. Hier zeigte sich, dass 48 % der Patienten innerhalb von 3 Jahren die Therapie gewechselt hatten. Eine unzureichende Wirksamkeit war die Hauptursache für den Wechsel. Ein Wechsel wurde häufiger bei Patienten beobachtet, die mit einer injizierbaren Erstlinientherapie behandelt wurden als bei Patienten mit Zweitlinientherapien wie Fingolimod oder Natalizumab. Eine Analyse an 595 deutschen MS-Patienten [74] mit einem Durchschnittsalter von 41,6 Jahren konnte zeigen, dass vor einem Wechsel mehr als 60 % der Patienten innerhalb von 12 Monaten ≥1 Schübe hatten. Die wesentlichen Gründe für den Wechsel der DMT waren das Versagen der aktuellen Therapie (53,9 %), der Wunsch des Patienten (22,4 %) und unerwünschte Ereignisse (19,0 %). Allerdings wurden trotz des nachweisbaren Optimierungsbedarfs nur 43,5 % der Patienten nachfolgend auf eine hochwirksame DMT umgestellt, obwohl Ärzte in der Mehrzahl Erwartungen an die klinischen und ergebnisorientierten Aspekte des Therapiewechsels äußerten. Die Daten belegen damit den noch immer zögerlichen Wechsel auf eine hochwirksame DMT, der wahrscheinlich den Sicherheitsaspekten dieser Substanzen geschuldet ist.

Dementsprechend sollte die Patientensicherheit und ein daran orientiertes Monitoring bei der Umstellung von DMTs beachtet werden [63]. Die Qualitätshandbücher des KKNMS haben in diesem Sinne Vorschläge gemacht, die im Wesentlichen dem Gedanken folgen, durch eine Therapiesequenz keine Sicherheitsprobleme zu generieren.

##### Empfehlung 10

- Der Wechsel von Dimethylfumarat, Glatirameracetat/Glatirameroiden und Interferonpräparaten auf eine andere DMT *sollte* ohne ein Auswaschintervall vollzogen werden. Auch hier ist allerdings – insbesondere bei Dimethylfumarat – eine Kontrolle des Differenzialblutbildes zu empfehlen und bei Lymphopenie ggf. bis zu einer Erholung des Blutbildes vor Umstellung zu warten.
- Bei Substanzen, die regelhaft zu einer Lymphopenie führen, wie Alemtuzumab, Cladribin oder S1P-Rezeptor-Modulatoren, *sollte* bis zu einer weitgehenden Rückbildung der Lymphopenie gewartet werden, wenn der klinische Zustand des Patienten eine Therapiepause erlaubt (Nutzen-Risiko-Analyse). Für konkrete Empfehlungen wird auf die Handbücher des KKNMS verwiesen.
- Bei Umstellung von Natalizumab auf eine andere hochwirksame Therapie *soll* das Risiko einer „Carry-over“-PML in Betracht gezogen werden. Daher *soll* vor Umstellung eine sorgfältige neuroradiologische Diagnostik erfolgen. Bei Bedarf *sollte *eine Analyse des Liquors auf HPyV-2(JCV)-DNA vorgenommen werden.
- Die potenziellen Wirkzeiten der DMTs *sollten* bei Umstellung in Betracht gezogen werden, insbesondere wenn Laborwerte oder sonstige organisatorische Erfordernisse eine Therapiepause bedingen. Die Wiederkehr von Krankheitsaktivität kann für MS-Patienten schwerwiegende Folgen haben und ist bei Natalizumab bereits nach der 6. Woche und bei S1P-Rezeptor-Modulatoren innerhalb der ersten 3 Monate nach Beendigung der Therapie möglich.

### (11) Welcher langfristige therapeutische Ansatz ist optimal zur Weiterbehandlung von Patienten, die zuletzt mit Alemtuzumab oder Cladribin behandelt wurden?

#### Review zu Evidenzen und entsprechende Empfehlungen

Depletierende Therapien mit Rekonstitution der Immunzellen stellen eine besondere Situation dar [73, 81]. Auch wenn die Mechanismen nicht genau geklärt sind, kommt es nach der Depletion mit Alemtuzumab oder Cladribin zu einer lang anhaltenden biologischen Wirkung, die über die Phase der Verminderung der Lymphozyten hinaus anhält. Die mechanistische Vorstellung ist, dass nach der Depletion das Immunsystem mit weniger autoimmunem Potenzial rekonstituiert wird. Beide Therapien werden nur über 2 Jahre mit 2 bzw. 4 Therapiezyklen verabreicht. Langzeitbeobachtungen zeigen, dass 50–60 % der Patienten nach Alemtuzumab oder Cladribin für 5 Jahre keine weitere Therapie benötigen und weitgehend frei von Schüben und Behinderungsprogression bleiben [20, 85]. Es existieren Berichte von Patienten mit über 10 Jahre anhaltender Stabilität. Bei dieser Patientengruppe ist das Vorgehen relativ einfach: Bei klinischer Stabilität muss zunächst keine weitere Immuntherapie erfolgen und lediglich der klinische Verlauf weiterverfolgt werden.

Eine andauernde Erkrankungsstabilität wird allerdings nicht bei allen Patienten beobachtet. Sollte im Verlauf erneute Krankheitsaktivität auftreten, so liegen keine Studien vor, welches Vorgehen am günstigsten ist. Zumindest für Alemtuzumab liegen Daten für einen 3. (sogar theoretisch 4. und 5.) Therapiezyklus vor [20, 114], die Aussicht auf Erfolg zur erneuten Stabilisierung der Erkrankungsaktivität demonstrieren, ohne das Risiko für Nebenwirkungen der Therapie zu erhöhen. Allerdings sind diese auch nicht geringer, sodass hier das Risiko einer Reapplikation besteht. Auch wenn prinzipiell alle anderen Folgetherapien denkbar sind, besteht bei den moderat wirksamen Therapien die Sorge einer nicht ausreichenden Wirksamkeit, da es sich bei den zuvor mit Alemtuzumab oder Cladribin Behandelten regelhaft um hochaktive Patienten handelt (siehe Empfehlungen zum Vorgehen nach [hoch]aktiven Therapien). Bei den stärker wirksamen DMTs könnte das Risiko für eine schwere Nebenwirkung erhöht sein, insbesondere aufgrund der lang anhaltenden biologischen Wirkung (nicht der Halbwertszeit) von Alemtuzumab bzw. Cladribin. Die sequenzielle Anwendung dieser beiden Therapeutika scheint daher nicht empfehlenswert zu sein, da die Summation der jeweils lang anhaltenden Effekte auf das Immunsystem nicht vorhersehbar ist. Alle anderen DMTs sind jedoch prinzipiell möglich, unter der Voraussetzung einer relativ engmaschigen Verlaufskontrolle bezüglich potenzieller schwerer Nebenwirkungen. Auch wenn dies in der Praxis umgesetzt wird bzw. werden muss, liegen keine systematisch erhobenen Daten zu solch einer sequenziellen Therapie vor.

Noch schwieriger ist die Situation, wenn es bereits im 1. Therapiejahr nach Gabe von Alemtuzumab bzw. Cladribin zu erneuter Krankheitsaktivität kommt. Dabei ist im Einzelfall nach Alemtuzumab sogar eine paradoxe Krankheitsaktivierung möglich [40, 121]. Andererseits ist Krankheitsaktivität im 1. Jahr nach Initiierung bei Alemtuzumab insbesondere nach Vorbehandlung mit Natalizumab oder S1P-Rezeptor-Modulatoren [88] nicht gänzlich ungewöhnlich. In den CareMS-Studien wurde bei Patienten mit inkomplettem frühem Ansprechen sogar ein besonders eindrucksvoller Effekt im 2. Jahr gesehen, sodass frühe Krankheitsaktivität nicht mit einem Therapieversagen gleichzusetzen ist. Soweit die Krankheitsaktivität zumindest teilweise vermindert ist (inkomplette frühe Therapieantwort), wäre es sinnvoll bzw. vertretbar, die Therapie im 2. Jahr erneut zu verabreichen, um das gewählte und langfristig angelegte Therapieprinzip zunächst fortzusetzen. Hiervon abzugrenzen sind Patienten mit ungebremster oder sogar gesteigerter Krankheitsaktivität (primäres Nichtansprechen oder paradoxe Erkrankungsexazerbation). Soweit die Entscheidung für eine frühe Umstellung der Therapie gefällt wird, dürften nur hochwirksame DMTs infrage kommen, da die Krankheitsaktivität unter einem hochwirksamen Medikament aufgetreten ist. Ein Wechsel von Cladribin auf Alemtuzumab bzw. umgekehrt erscheint aufgrund der unklaren Folgen für die Immunkompetenz auch hier eher nicht ratsam (siehe oben). Somit kommen vorrangig Anti-CD20-Antikörper, Natalizumab oder S1P-Rezeptor-Modulatoren infrage, wobei die jeweiligen Vortherapien in die individuelle Entscheidung einfließen.

##### Empfehlung 11

- Sollte es nach den komplett durchgeführten Therapiezyklen mit Alemtuzumab zu erneuter Krankheitsaktivität im Verlauf kommen, *sollte* ein 3. Therapiezyklus erwogen werden, wenn es nach den ersten Zyklen zu einer deutlichen Reduktion der Krankheitsaktivität gekommen ist (Jahr 2) und damit von Therapieansprechen bzw. Krankheitskontrolle auszugehen ist.
- Sollte es nach den komplett durchgeführten Therapiezyklen mit Cladribin zu erneuter Krankheitsaktivität im Verlauf kommen, *kann* ein 3. Therapiezyklus analog zu Alemtuzumab erwogen werden (für Cladribin im 3. und 4. Jahr derzeit „off-label“), allerdings ist hierfür keine ausreichende Datenlage verfügbar.
- Ein Wechsel von Alemtuzumab zu Cladribin oder umgekehrt *sollte* aufgrund der wenig absehbaren, sequenziell kombinierten Wirkung auf das Immunsystem gut begründet sein. Alle anderen zugelassenen DMTs *können* prinzipiell unter engmaschiger Kontrolle bei Auftreten von Krankheitsaktivität verabreicht werden.
- Ist unter der Therapie mit Alemtuzumab oder Cladribin im 1. Therapiejahr Krankheitsaktivität zu verzeichnen, so *sollte* dies einerseits im Kontext der Vortherapie bzw. der Erkrankungsvorgeschichte (vorbestehende Aktivität/Schwere) interpretiert werden, andererseits *sollte* – sofern die Erkrankung nicht gleichbleibend aktiv oder sogar paradox aktiver geworden ist – der Effekt dieses Therapieprinzips mit Gabe des kompletten weiteren Therapiezyklus abgewartet werden, um das Therapieprinzip zu wahren. Ist die Krankheitsaktivität im Vergleich zur Status vor Therapieinitiierung nach dem 1. Therapiezyklus allerdings klar verstärkt, *sollte* die Umstellung auf eine andere (hoch)aktive DMT vorgenommen werden.

### (12) Wie sollte die therapeutische Strategie bei Patienten mit MS, die mit DMTs behandelt werden, aussehen, um das Risiko einer progressiven multifokalen Leukenzephalopathie (PML) zu minimieren?

#### Review zu Evidenzen und entsprechende Empfehlungen

Allen DMTs ist aufgrund ihrer Wirkung auf das Immunsystem ein grundsätzliches Risiko für immunologische Nebenwirkungen zuzusprechen, zumal keine der verfügbaren Therapien selektiv oder spezifisch auf die MS wirkt.

Eine Reihe von Studien hat sich in den vergangenen Jahren mit dem Risiko für Infektionen und immunologische Nebenwirkungen von DMTs auseinandergesetzt (z. B. [72]). Aufgrund der Tatsache, dass insbesondere Sicherheitsbedenken immunologischer Art die breite Anwendung hochwirksamer Therapien limitieren, ist ein wesentliches Ziel des Monitorings von DMTs die Vermeidung sowie die Früherkennung und das Management von Nebenwirkungen, hier insbesondere von Infektionen (z. B. [63]). Besonders prominent ist das Risiko einer PML durch HPyV‑2 (JCV) im Zusammenhang mit DMTs [8, 65, 105]. Während die Behandlung mit Natalizumab zweifelsohne das höchste Risiko für eine therapieassoziierte PML mit sich bringt, ist dies jedoch nicht die einzige Substanz, bei der PML-Fälle beschrieben wurden, sodass gegenwärtig Medikamente mit hohem Risiko (>1:1000: Natalizumab), mit mittlerem Risiko (1:1000–1:50.000: Dimethylfumarat, Fingolimod, Ozanimod, Siponimod) sowie solche mit niedrigem Risiko (<1:50.000: Alemtuzumab, Cladribin, Glatirameracetat/Glatirameroide, Interferone, Ocrelizumab, [Rituximab], Teriflunomid) unterschieden werden [8, 63, 65]. Hinsichtlich der Risikostratifizierung für eine PML unter Natalizumab haben die vergangenen Jahre wesentliche Erkenntnisse gebracht: eine Stratifizierung nach bestimmten Parametern, zu denen i) die Therapiedauer, ii) eine vorhergehende Immunsuppression, iii) das Vorhandensein von anti-HPyV-2(JCV)-Antikörpern (AK) und die Quantität derselben (HPyV-2[JCV]-AK-Index) determinierend für das kalkulierte Risiko für die Entwicklung einer PML sind (rangierend von <1:10 bis >1:10.000; [48, 90, 105]). Eine Reihe anderer Marker sind in der Entwicklung, allerdings bislang in der Praxis entweder nicht zugänglich oder verfügbar (z. B. CD62L-Titer, lipidspezifische IgM-Banden). Das Risiko für eine PML steigt letztlich abhängig von der Dauer der Therapie, also mit jeder Natalizumabinfusion. Deswegen müssen Patienten unter Natalizumabtherapie kontinuierlich evaluiert werden, bei negativem HPyV-2(JCV)-AK Status wird die Bestimmung mittels ELISA alle 6 Monate empfohlen. HPyV-2(JCV)-AK-positive Patienten mit einem Index von ≥0,9 sollten grundsätzlich nicht länger als 18 Monate mit Natalizumab behandelt werden. Ausnahmen sind möglich, wenn die Therapien engmaschig sowohl klinisch (alle 3 Monate) sowie mittels einer MRT (alle 3 bis 6 Monate), ggf. mit einem Spezialprogramm zum Auffinden von Symptomen einer PML, überwacht werden. Die MRT-Beurteilung erfolgt mittels diffusionsgewichteter Bildgebung (DWI) und Postkontrastsequenzen [119].

Das Risiko für eine Serokonversion unter Natalizumab scheint etwas höher als das der Durchschnittspopulation und das von MS-Patienten generell (2–10 %/Jahr, [104, 106, 112]).

Als eine weitere Maßnahme zur Minderung des PML-Risikos wurde jüngst das „extended interval dosing“ (EID) propagiert, hauptsächlich basierend auf der Analyse aus dem TOUCH-Register [100]. Diese retrospektive Analyse ergab, dass ein verlängertes Dosierungsintervall (durchschnittlich 6 Wochen vs. 4 Wochen laut initialer Zulassung) zu einem signifikant niedrigeren PML-Risiko führt als eine Therapie mit dem zugelassenen Dosierungsintervall. Das Ergebnis wird von den Autoren als Klasse-III-Evidenz eingeordnet, da es sich um eine retrospektive Datenauswertung handelt. Daten aus einer prospektiven Studie, in der die Frage der Wirksamkeit eines 6‑wöchigen vs. 4‑wöchigen Dosierungsintervalls adressiert werden (NOVA-Studie), liegen in vorläufiger Form vor und deuten – nicht unerwartet – auf den Erhalt der Wirksamkeit auch bei verlängertem Dosisintervall [17]. Eine sichere Handlungsempfehlung kann zum gegenwärtigen Zeitpunkt daraus nicht abgeleitet werden, wenn auch die Studie und die Möglichkeit eines verlängerten Dosisintervalls in der Fachinformation genannt werden (Verweis Fachinformation Natalizumab). Unklar ist momentan ebenfalls, wie sich die neu zugelassene subkutane Applikationsform von Natalizumab hinsichtlich des PML Risikos einordnen lässt.

Bei den weiteren Medikamenten ist Dimethylfumarat insofern erwähnenswert, als dass die Überwachung der Leukozytenwerte und die Anzahl der absoluten Lymphozyten eine Vorgabe ist, die zur prospektiven Vermeidung von Sicherheitsrisiken, inklusive PML, angeraten ist. Dimethylfumarat reduziert bei ca. 10 % der Patienten die Zahl der Lymphozyten und Leukozyten signifikant, bei bestätigten Werten von <500/µl ist die Substanz abzusetzen. Im Graubereich persistenter Lymphopenien 2. Grades (500–800/µl) sind höherfrequente Überwachungen angeraten, zumal auch PML-Fälle bei Lymphozytenwerten im Bereich des 2. Grades aufgetreten sind. Hier ist das Alter der Patienten auf Basis von Immunseneszenzprozessen wahrscheinlich ein wichtiger Kofaktor für die Entstehung der PML. Insgesamt betrachtet liegt das Risiko einer PML im Zusammenhang mit Dimethylfumarat bei ca. 1:45.000.

Das Risiko einer PML während der Therapie mit Fingolimod liegt bei knapp unter 1:10.000 (nicht kalkuliert sind hier die „Carry-over“-PML-Fälle von Natalizumab zu Fingolimod). Trotzdem gibt es keine echte Maßnahme oder Laborwerte zur Stratifizierung des PML-Risikos, außer des in Deutschland üblichen Lymphozytengrenzwerts von 200/µl, der nicht kontinuierlich unterschritten werden soll. Es darf allerdings bezweifelt werden, dass die Zahl der peripheren Lymphozyten mit dem Risiko für eine PML korreliert, zumal auch die Rate der generellen Infektionen offenbar nicht mit der Zahl der Lymphozyten korreliert. Aufgrund des Wirkmechanismus ist davon auszugehen, dass andere S1P-Rezeptor-Modulatoren ebenfalls das Risiko einer PML – neben anderen klassenspezifischen immunologischen Nebenwirkungen – mit sich bringen.

Für die anderen Medikamente (Alemtuzumab, Cladribin, Ocrelizumab, Ofatumumab, Teriflunomid) besteht gegenwärtig die Ansicht, dass zwar grundsätzlich ein PML-Risiko besteht, dieses aber zu niedrig ist, um eine echte Risikostratifizierung vorzuschlagen. Wie oben beschrieben, scheint insbesondere das Alter ein wesentlicher Prädispositionsfaktor zu sein (hierauf deuten auch die 2 bislang – Stand 04/2021 – unter Ocrelizumab aufgetretenen PML-Fälle hin).

##### Empfehlung 12

- Natalizumab ist i) bei positivem HPyV-2(JCV)-AK-Status (≥0,9) und ii) einer Behandlungsdauer von ≥18 Monaten mit einem hohen Risiko für die Entwicklung einer PML verbunden. Unabhängig vom HPyV-2(JCV)-AK-Status ist iii) eine vorhergehende Immunsuppression ein Risikofaktor, der zusammen mit der Behandlungsdauer berücksichtigt werden soll.
- Bei stabilem Verlauf *sollen* Patienten mit niedrigem Risiko (HPyV-2[JCV]-AK negativ, HPyV-2[JCV]-AK-Titer ≤0,9) zur Überwachung der Behandlungssicherheit regelmäßige klinische Untersuchungen erhalten und mindestens einmal jährlich paraklinisch (MRT) untersucht werden. Auch *sollen* HPyV-2(JCV)-AK-Titer-Kontrollen alle 6 Monate erfolgen.
- Bei Patienten mit einem hohen PML-Risiko (HPyV-2[JCV]-AK-Titer ≥0,9 und Dauer der Behandlung mit Natalizumab >18 Monate, vorhergehende Immunsuppression) *sollen* im Fall einer Fortsetzung der Therapie häufigere MRT-Kontrollen (alle 3 bis 6 Monate) vorgenommen werden. Parallel dazu *sollen* zudem höherfrequente klinische Kontrollen (≤ alle 3 Monate) erfolgen. Hier ist die Durchführung von MRT-Kurzprotokollen mit speziellen PML-Sequenzen möglich.
- Für Patienten mit einem hohen PML-Risiko, die das Medikament wechseln, oder bei Patienten mit einem Potenzial für erhöhte Krankheitsaktivität nach Absetzen der Therapie – wie beim Wechsel von Natalizumab auf Fingolimod – *sollen* insbesondere zum Zeitpunkt des Absetzens der aktuellen Behandlung und nach Beginn der neuen Behandlung eine zerebrale MRT und ggf. eine Liquoruntersuchung erfolgen (HPyV-2[JCV]-PCR), um eine „Carry-over“-PML sicher auszuschließen und die Situation zum Therapieende bzw. nach Therapieinitiierung zu erheben.
- Zur Vermeidung immunologischer Nebenwirkungen – inklusive opportunistischer Infektionen wie PML – soll insbesondere im 1. Jahr unter Dimethylfumarat eine Überwachung der Leukozyten und speziell der absoluten Lymphozyten erfolgen. Sollte die Zahl persistent unter 500/μl liegen, muss das Präparat abgesetzt werden. Bei anhaltenden Werten zwischen 500 und 800/μl – auch über das 1. Jahr hinaus – sind verstärkte Vigilanzmaßnahmen anzuwenden (höherfrequentes klinisches und magnetresonanztomographisches Monitoring). Das Risiko steigt offenbar mit dem Alter der Patienten (≥55 Jahre).
- Für das PML-Risiko unter Fingolimod (und vermutlich den anderen S1P-Modulatoren) gibt es außer einem höheren Patientenalter (≥55 Jahre) keine Risikostratifizierungsparameter.

### (13) Für MS-Patientinnen unter einer DMT: Wie ist die Therapie bei Kinderwunsch oder einer ungeplanten Schwangerschaft zu verändern und wie sollte die Behandlung nach der Entbindung während der Stillzeit aussehen?

#### Review zu Evidenzen und entsprechende Empfehlungen

Mit der steigenden MS-Inzidenz bei Frauen hat das Thema eines Kinderwunsches unter Therapie weiter an Bedeutung gewonnen. Eine gute Übersicht hierzu gibt Montalban et al. (2018) [78]. Da naturgemäß keine prospektiven Studien existieren, gibt es zu DMTs mit lange bestehender Zulassung die größte Erfahrung: Glatirameracetat/Glatirameroide und Interferon-β-Präparate sind grundsätzlich während der Schwangerschaft zugelassen, wobei von ärztlicher Seite die Indikation zum Nutzen einer Therapiefortführung gestellt werden sollte [41, 45]. Auch wird man generell bei hochaktiven Formen der MS jungen Frauen zunächst zur Stabilisierung der Erkrankung raten, bevor eine Schwangerschaft angestrebt wird. Sollte bei diesen Patientinnen trotzdem ungeplant eine Schwangerschaft eintreten, kann die Fortführung von Natalizumab erwogen werden. Die Therapie sollte dann ab der 32. SSW bis zur Entbindung unterbrochen werden, da es bei längerer Behandlung zu Blutbildveränderungen beim Kind kommen kann.

Bedenken gibt es naturgemäß bei DMTs, die immunsuppressiv in die DNA-Replikation eingreifen oder Moleküle tangieren, die bei der intrauterinen Entwicklung entscheidend sind. Bei Konzeption unter Teriflunomid soll das Medikament forciert durch Cholestyramin eliminiert werden. Unter S1P-Modulatoren ist – basierend auf Erfahrungen mit Fingolimod – wegen der Gefahr schwerer Fehlbildungen eine Konzeption zu vermeiden. Dabei kann eine Differenzierung zwischen Substanzen mit kurzer (Siponimod, Ponesimod) vs. langer Eliminationszeit (Fingolimod, Ozanimod) relevant sein. Auch der Einsatz von Cladribin verbietet sich während der Schwangerschaft. Depletierende Antikörper können während der Schwangerschaft nur nach strenger Nutzen-Risiko-Abwägung eingesetzt werden. Bei letzteren besteht kein bekanntes Fehlbildungsrisiko, allerdings kann es insbesondere im letzten Trimenon zu immunologischen Effekten beim Kind kommen. Aufgrund der langen immunologischen Wirkdauer eignen sich Cladribin und Alemtuzumab (insbesondere nach einem vollständigen Behandlungszyklus) sowie Anti-CD20-Antikörper, um die Konzeption während der Therapiefreiheit zu versuchen (siehe Empfehlungen).

Anzufügen bleibt, dass bei der breit eingesetzten oralen DMT Dimethylfumarat schon eine große Post-Marketing-Erfahrung besteht und dass keine teratogenen Effekte beobachtet wurden [110].

##### Empfehlung 13

- Patientinnen *sollen* darüber informiert werden, dass mit Ausnahme von Interferonen und Glatirameracetat/Glatirameroiden die DMTs während der Schwangerschaft keine Zulassung haben.
- Gleiches gilt für die Stillzeit, für die bisher nur für Interferone eine Zulassung besteht.
- Für Frauen mit hoher Krankheitsaktivität *sollte* die Kontrolle der Erkrankungsaktivität prioritären Stellenwert besitzen, sodass man in diesen Fällen zunächst eine Verschiebung einer geplanten Schwangerschaft anrät.
- Für geplante oder ungeplante Schwangerschaften bei hoher Krankheitsaktivität *kann* Natalizumab bis zur 32. Woche gegeben werden, unter klarer Besprechung der Nutzen-Risiko-Abwägungen.
- Die Gabe von Dimethylfumarat *kann* bis zum positiven Schwangerschaftstest erwogen werden.
- Die Behandlung mit immundepletierenden DMTs (Alemtuzumab, Cladribin) *kann* bei bestehendem Kinderwunsch eine alternative therapeutische Option sein, sofern das Intervall von letzter Gabe bis zur Konzeption ≥4 (Alemtuzumab) bzw. ≥6 Monate (Cladribin, für beide Geschlechter) ist.
- Aus der Kenntnislage der klinischen Routine *ist es möglich* ≥4 Monate nach einer Behandlung mit Ocrelizumab eine Konzeption zu planen („good clinical practice point“; siehe auch Qualitätshandbücher des KKNMS).
- Für Frauen mit hohem Risiko für eine Erkrankungsaktivierung *sollen* Interferone oder Glatirameracetat/Glatirameroiden als Therapie bis zum Eintritt oder auch während der Schwangerschaft in Erwägung gezogen werden.
- Grundsätzlich *sollte* eine Immuntherapie nach Entbindung, unter Berücksichtigung der Vorgaben und Einschränkungen in der Stillzeit, wieder aufgenommen werden.

### (14) Wie sollte die Behandlungsstrategie im aktuellen epidemiologischen Kontext von COVID-19 aussehen?

#### Review zu Evidenzen und entsprechende Empfehlungen

Die Pandemie mit SARS-CoV‑2 im Jahr 2020 hat den Fokus auf die Frage gelegt, inwieweit DMTs die Reaktion des Immunsystems gegen neue (virale) Antigene beeinträchtigen. Zum jetzigen Zeitpunkt können diese Fragen zwar noch nicht abschließend beantwortet werden, die bereits verfügbaren Daten aus Registerstudien erlauben jedoch eine erste Positionierung, nachdem in der Frühphase der Pandemie bereits grundsätzliche Empfehlungen hinsichtlich des Zusammenhangs MS – Infektionen – Umgang mit Immuntherapie gegeben wurden (exemplarisch [66]).

So konnten die Daten von mehr als 300 Patienten des französischen COVISEP-Registers im Hinblick auf das Outcome der SARS-CoV-2-Infektion auch bei Exposition mit unterschiedlichen DMTs ausgewertet werden [69]. Die Autoren konnten zeigen, dass das Risiko für einen schweren COVID-19-Verlauf bei MS-Patienten mit dem Alter und dem Behinderungsgrad (EDSS) zunimmt. Die multivariate Regressionsanalyse bestätigte ein höheres Lebensalter, einen höheren EDSS, kardiale Vorerkrankungen und Adipositas als Risikofaktoren für einen schweren Verlauf – also somit ähnliche Risikofaktoren wie in der nicht von MS betroffenen Allgemeinbevölkerung. Zwischen den Patientengruppen mit unterschiedlichen DMTs wurden in dieser Auswertung keine Unterschiede hinsichtlich des COVID-19-Verlaufes festgestellt, ungünstigere Verläufe fanden sich hingegen eher bei unbehandelten Patienten. Es muss allerdings in Betracht gezogen werden, dass sich in der Gruppe der unbehandelten Patienten vermehrt ältere MS-Patienten mit progredienten Verläufen befanden.

Die Auswertung des italienischen Musc-19-Registers kam hingegen zu dem Schluss, dass bei insgesamt guter Sicherheit der DMTs (auch nach Adjustierung für Alter und Verlauf der Erkrankung) ein erhöhtes Risiko für schwere COVID-19-Verläufe in der Subgruppe der mit Anti-CD20-mAb behandelten Patienten sowie nach Kortikosteroidtherapie festgestellt wurde. Ähnliche Schlüsse wurden auch bei der Auswertung der Daten der sog. Global Data Sharing Initiative gezogen, einer weltweiten Datensammlung von MS-Patienten mit COVID-19 [86], die die Endpunkte Krankenhauseinweisung, Aufnahme auf eine Intensivstation, Beatmung und Tod untersuchte. Wie in anderen Registerstudien war das Erreichen dieser Endpunkte mit einem höheren Lebensalter, dem Vorliegen einer progredienten MS-Verlaufsform und mit EDSS-Werten von >6 assoziiert. In Bezug auf die MS-Therapien konnten die Autoren feststellen, dass neben unbehandelten Patienten auch Patienten, die mit einer B‑Zell-depletierenden Therapie behandelt wurden, ein höheres Risiko für einen schwereren COVID-19-Verlauf hatten. Bei genauer Betrachtung der Daten gilt dies aber nicht für den tödlichen Ausgang der Infektion, hier zeigte sich kein signifikanter Unterschied zwischen den Anti-CD20- und anderen MS-Therapien. Ein nordamerikanisches Register analysierte 1626 Patienten und konnte zeigen, dass ein höherer Behinderungsgrad mit schlechterem COVID-19-Verlauf assoziiert war; dies galt auch für Alter, kardiovaskuläre Komorbiditäten, rezente Behandlung mit Kortikosteroiden sowie Rituximab [103].

Neben den Fragen zu den DMTs hat die Corona-Pandemie auch die Frage nach der optimalen Impfstrategie für MS-Patienten aufgeworfen (z. B. 10.1177/13524585211003476). Hier existieren verständlicherweise noch keine ausreichenden Daten für MS-Patienten, da die Impfungen gerade erst angelaufen sind. Daher müssen die Empfehlungen aus den allgemeinen Erfahrungen zum Impfen bei immunsuppressiver Therapie abgeleitet werden [117]. Demnach können Totimpfstoffe grundsätzlich gefahrlos verabreicht werden, vorzugsweise sollte die Impfung 2 bis 4 Wochen vor Beginn einer immunsuppressiven Therapie abgeschlossen sein. Lebendimpfstoffe sind grundsätzlich bei Patienten mit immunsuppressiver Therapie kontraindiziert bzw. bedürfen einer sorgfältigen Nutzen-Risiko-Abwägung. Der Zeitraum zwischen Lebendimpfung und Beginn einer immunsuppressiven Therapie sollte mindestens 6 Wochen betragen. Daraus ergibt sich, dass bei MS-Patienten grundsätzlich vor Beginn einer Therapie der Impfstatus überprüft werden und gemäß entsprechender Vorgaben aktualisiert werden sollte. Oft ist dies aber vor Beginn der MS-Therapie versäumt worden bzw. wie aktuell im Falle der Impfung gegen SARS-CoV‑2 organisatorisch nicht möglich. In diesem Fall stellt sich die Frage nach dem optimalen Timing der Impfung unter der jeweiligen Therapie. Es muss bedacht werden, dass einige DMTs die Reaktionsbereitschaft des Immunsystems herunterregulieren und dadurch eine Impfung theoretisch nicht zu der gewünschten und ausreichenden Schutzimmunität führen kann. Dieses Problem ist bei bestimmten DMTs, wie Dimethylfumarat, Glatirameracetat/Glatirameroiden, Interferonpräparaten, Natalizumab oder Teriflunomid eher gering ausgeprägt, bei Cladribin, Fingolimod oder Ozanimod wahrscheinlich stärker. Bei den DMTs, deren Wirkmechanismus auf der Depletion der insbesondere für die Impfantwort wichtigen B‑Zellen beruht (Alemtuzumab, Ocrelizumab, Ofatumumab), könnten allerdings aus abgeleiteten Erwägungen Probleme auftreten. Hierbei ist zu beachten, dass das bislang nach ca. 6 Monaten gemessene immunologische Gedächtnis gegen SARS-CoV‑2 offensichtlich keine Korrelation zwischen T‑Lymphozyten und B‑Zellantworten zeigt [28] und der Immunitätsschutz nicht nur über Antikörperbildung, sondern auch über T‑Lymphozyten (CD4 sowie vor allem CD8) vermittelt wird, sodass die Messung der Antikörperbildung letztlich nicht allein aussagekräftig für den erwarteten Schutz vor Infektion (oder schwererem Verlauf) ist.

Für einige der verfügbaren DMTs wurden bereits Studien zur Wirkung anderer Impfungen durchgeführt [4, 5, 57]. Es zeigte sich bei einigen Medikamenten zwar grundsätzlich eine etwas reduzierte Impfantwort, die aber in der Mehrzahl für den Impfschutz ausreichend war. Von besonderem Interesse ist hier die Studie zur Impfantwort 12 Wochen nach Gabe von Ocrelizumab (VELOCE, [4]). Hier betrug die positive Ansprechrate auf den Tetanusimpfstoff nach 8 Wochen 23,9 % in der Ocrelizumabgruppe gegenüber 54,5 % in der Kontrollgruppe (wobei als positive Ansprechrate die ≥4-fache Zunahme des Antikörpertiters durch die Impfung definiert wurde – eine Serumprotektion nach Titerdefinition haben erfreulicherweise alle Probanden der Ocrelizumab und der Kontrollgruppe erreicht). Die positive Ansprechrate auf ≥5 Serotypen des polyvalenten Pneumokokkenimpfstoffes nach 4 Wochen betrug 71,6 % in der Ocrelizumabgruppe und 100 % in der Kontrollgruppe. Die Ansprechraten nach 4 Wochen gegen die 5 verimpften Influenzastämme lagen in der Ocrelizumabgruppe zwischen 55,6 und 80,0 % und in der Kontrollgruppe zwischen 75,0 und 97,0 %. Gegen ein echtes „Neoantigen“ wie KLH waren praktisch keine Antikörperantworten nachweisbar.

##### Empfehlung 14

- Auf der Basis der bisher verfügbaren Daten stellen Komorbiditäten und kritische Residuen der MS mit schwerer Behinderung ein erhöhtes Risiko für einen schweren Verlauf von COVID-19 dar, nicht aber die MS-Erkrankung selbst oder eine der DMTs. Zu CD20-Antikörpern existieren allerdings Daten, die das Risiko eines möglicherweise schwereren Verlaufs zeigen. Es gibt demnach keinen Grund, jüngeren und ansonsten gesunden MS-Patienten eine Therapie aufgrund der Pandemie vorzuenthalten bzw. eine Therapie zu verschieben. Die Auswahl eines Medikamentes sollte sich weiterhin ausschließlich nach der Aktivität und Schwere der MS richten.
- Bei MS-Patienten mit höherem Lebensalter, höherem Behinderungsgrad und internistischen, insbesondere kardiovaskulären Vorerkrankungen *sollte *allerdings zu Zeiten der Pandemie intensiver die Erkrankungsaktivität in Trajektorie zum Alter reflektiert werden, insbesondere bei Indikation progredienter MS, und ob eine MS-Therapie/B-Zell-depletierende Immuntherapie wirklich indiziert ist. Eine Kortikosteroidtherapie kann das Risiko für schwerere Verläufe erhöhen, was bei Patienten mit regelmäßig durchgeführten Kortikosteroidpulsen zu bedenken ist.
- Grundsätzlich *sollen* MS-Patienten geimpft werden – dies schließt die Impfung gegen SARS-CoV‑2 mit ein und gilt für alle gegenwärtig verfügbaren (konzeptuellen Tot‑)Impfstoffe (mRNA, Adenovirusvektor). Auch unter einer DMT sollte die Impfung durchgeführt werden; es besteht auch bei einer laufenden DMT eine berechtigte Aussicht darauf, dass eine suffiziente Impfantwort erreicht wird. Daher wird auch bei den meisten DMTs kein besonderes Timing hinsichtlich der Impfung benötigt. Bei Ocrelizumab existieren Daten zur Impfung 3 Monate nach Gabe der Therapie, daher *kann* dieses Zeitfenster, wenn möglich, beachtet werden. Wenn dies nicht möglich ist, *kann* im Sinne der Nutzen-Risiko-Abwägung wann immer möglich gegen SARS-CoV‑2 geimpft werden (siehe Merkblatt Impfen KKNMS Stand 04.03.2021). Ansonsten *soll* den Empfehlungen der jeweiligen nationalen Impfkommission Folge geleistet werden. Zur Überprüfung der Impfantwort durch Messung von Antikörpern sind zurzeit keine ausreichenden Daten vorhanden und dieses wird auch von den Herstellern nicht empfohlen.

## Caption Electronic Supplementary Material


