# Supplementary material for: Multiple sclerosis treatment consensus group (MSTCG): position paper on disease-modifying treatment of multiple sclerosis 2021 (white paper)
Source: Nervenarzt. 2021 Jul 23;92(8):773–801. [Article in German] doi: 10.1007/s00115-021-01157-2 (PMC8300076; doi:10.1007/s00115-021-01157-2)
Supplement: Supplementary file 1 [file 115_2021_1157_MOESM1_ESM.docx]

*Interessenkonflikte*

HW: Erhielt Zuschüsse oder Verträge von BMBF, Novartis, Genzyme, Roche, Merck, Biogen, DFG, Europäische Union; Tantiemen oder Lizenzen von Hogrefe, Springer Medizin; Beratungshonorare von Merck, Novartis, Roche, Sanofi; Bezahlung für Expertenaussagen von AXA banque patrimoniale, Dialectica, Schweizer MS-Gesellschaft, Peervoice; Unterstützung für die Teilnahme an Meetings und/oder Reisen von Biogen, FEO, Merck, Novartis, Pfizer, Sanofi, Celgene, Excell/Bial, Roche. Teilnahme an einem Data Safety Monitoring Board oder Advisory Board für PSI CRO Deutschland.

RG: Erhielt Zuschüsse oder Verträge von Novartis, Biogen, Sanofi; Beratungshonorare von Merck-Serono, Janssen, Biogen, Novartis, Sandoz; Zahlungen oder Honorare für Vorträge, Präsentationen, Rednerbüros, Manuskriptschreiben oder Bildungsveranstaltungen von BayerVital, Biogen, Eisai, Bristol Myers Squibb, TEVA, Merck-Serono, Novartis, Roche, Sanofi-Aventis. Teilnahme an einem Data Safety Monitoring Board oder Advisory Board für Roche, Genentech; Führungs- oder Treuhandfunktion in einem anderen Gremium, einer Gesellschaft, einem Komitee oder einer Interessengruppe, bezahlt oder unbezahlt als Vorsitzender des DMSG Medical Advisory Board (Ehrenamt). Hat Aktien oder Aktienoptionen für Roche, Bayer, Merck.

TB: Erhielt Zuschüsse oder Verträge von der Österreichischen MS-Gesellschaft, dem Österreichischen Forschungsfonds, der Österreichischen Nationalbank, der Österreichischen Gesellschaft für Neurologie und von Alexion, Biogen, Merck, Novartis, Roche Sanofi-Genzyme; Beratungshonorare von Biogen, Bionorica, MedDay, Merck, Novartis, Roche, Sanofi-Genzyme; Zahlungen oder Honorare für Vorträge, Präsentationen, Rednerbüros, Manuskriptschreiben oder Bildungsveranstaltungen von Allergan, Biogen, Biologix, Bionorica, Celgene/BMS, MedDay, Merck, Novartis, Roche, Sanofi-Genzyme, TEVA. Führende oder treuhänderische Rolle in einem anderen Gremium, einer Gesellschaft, einem Komitee oder einer Interessengruppe, bezahlt oder unbezahlt als Präsident der Österreichischen Gesellschaft für Neurologie (unbezahlt), Präsident der Österreichischen MS-Forschungsgesellschaft (unbezahlt), Vorsitzender des wissenschaftlichen Komitees und Vorstandsmitglied der Europäischen Akademie für Neurologie (unbezahlt).

TD: Erhielt Zuwendungen oder Verträge von Alexion, Biogen, Roche; Beratungshonorare von Alexion, Biogen, Celgene, Geneuro, Novartis, Roche, Sanofi, Merck; Zahlungen oder Honorare für Vorträge, Präsentationen, Rednerbüros, Manuskriptschreiben oder Bildungsveranstaltungen von Alexion, Biogen, Celgene, Novartis, Roche, Sanofi, Merck. Teilnahme an einem Data Safety Monitoring Board oder Advisory Board für Roche, Polyneuron, MedDay, Alexion, Biogen, Celgene, Novartis, Roche, Sanofi, Merck. Leitende oder treuhänderische Rolle in einem anderen Gremium, einer Gesellschaft, einem Komitee oder einer Interessengruppe, bezahlt oder unbezahlt als Vorstandsmitglied von ECTRIMS (unbezahlt). Andere finanzielle oder nicht-finanzielle Interessen: meine Frau ist Mitarbeiterin von Novartis und hält Aktien von Novartis.

RL: Erhielt Zuschüsse oder Verträge von Novartis; Beratungshonorare von Biogen, Celgene/BMS, Janssen, Merck, Novartis, Roche; Zahlungen oder Honorare für Vorträge, Präsentationen, Rednerbüros, Manuskriptschreiben oder Bildungsveranstaltungen von Celgene/BMS, Merck, Novartis, Roche, Sanofi-Genzyme. Teilnahme an einem Data Safety Monitoring Board oder Advisory Board für Novartis, Celgene; Führungs- oder Treuhandfunktion in einem anderen Gremium, einer Gesellschaft, einem Komitee oder einer Interessengruppe, bezahlt oder unbezahlt als Beisitzer Vorstand KKMMS, Stv. Vorsitz ÄB DMSG.

MM: Erhielt Zuschüsse oder Verträge von Merck; Beratungshonorare von Biogen, Bayer, Jansen, Celgene, Merck, Novartis, Roche, Sanofi-Genzyme; Zahlungen oder Honorare für Vorträge, Präsentationen, Rednerbüros, Manuskriptschreiben oder Bildungsveranstaltungen von Biogen, Bayer, Jansen, Celgene, Merck, Novartis, Roche, Sanofi-Genzyme.

MS: Erhielt Zuschüsse oder Verträge von der DFG und der Volkswagenstiftung sowie von Merck-Serono, Novartis, Sanofi-Genzyme; Beratungshonorare von Alexion, Biogen, Celgene, CSL Behring, Grifols, Janssen, MedDay, Merck-Serono, Novartis, Roche, Sanofi-Genzyme, Teva; Zahlungen oder Honorare für Vorträge, Präsentationen, Rednerbüros, Manuskriptschreiben oder Bildungsveranstaltungen von Alexion, Biogen, Bayer Vital, Celgene, CSL Behring, Janssen, Merck-Serono, Novartis, Roche, Sanofi-Genzyme, Takeda, Teva. Teilnahme an einem Data Safety Monitoring Board oder Advisory Board für Takeda; Führungs- oder Treuhänderrolle in einem anderen Gremium, einer Gesellschaft, einem Komitee oder einer Interessengruppe, bezahlt oder unbezahlt als Vorstandsmitglied Deutsche Gesellschaft für Neurologie (DGLN) (unbezahlt), Mitglied im Beirat Deutsche MS-Gesellschaft (DMSG) (unbezahlt).

OA: Erhielt Zuschüsse oder Verträge von Biogen, Merck, Novartis, Roche; Beratungshonorare von Biogen, Celgene, Merck, Novartis, Roche; Zahlungen oder Honorare für Vorträge, Präsentationen, Rednerbüros, Manuskripterstellung oder Bildungsveranstaltungen von Almirall, Biogen, Merck, Novartis, Roche, Sanofi, Teva; Unterstützung für die Teilnahme an Tagungen und/oder Reisen von Biogen, Merck, Roche.

KB: Erklärt, dass kein Interessenkonflikt besteht.

MB: Erhielt Zuschüsse oder Verträge von Novartis; Beratungshonorare von Sanofi-Aventis Dtl. GmbH, Novartis Pharma GmbH, Janssen-Cilag GmbH, Roche Pharma AG, Merck Serono GmbH, Biogen GmbH; Zahlungen oder Honorare für Vorträge, Präsentationen, Rednerbüros, Manuskriptschreiben oder Bildungsveranstaltungen von Sanofi-Aventis Dtl. GmbH, Novartis Pharma GmbH, Roche Pharma AG, Alexion Pharma GmbH, Merck Serono GmbH; Unterstützung für die Teilnahme an Sitzungen und/oder Reisen von Celgene GmbH, Biogen GmbH, Novartis Pharma GmbH.

SB: Erhielt Zahlungen oder Honorare für Vorträge, Präsentationen, Rednerbüros, Manuskriptschreiben oder Bildungsveranstaltungen von Biogen Idec, Bristol Meyer Squibbs, Merck Serono, Novartis, Roche und Sanofi Genzyme; Unterstützung für die Teilnahme an Meetings und/oder Reisen von Merck Serono.

ACh: Erhielt Zuschüsse oder Verträge von der EU (Horizon 2020 research grant), EU (Marie-Curie network initiative), Schweizer Nationalstiftung, Schweizerische MS-Gesellschaft; Zahlungen oder Honorare für Vorträge, Präsentationen, Rednerbüros, Manuskriptschreiben oder Bildungsveranstaltungen von Actelion-Janssen, Almirall, Bayer, Biogen, Celgene, Sanofi-Genzyme, Merck, Novartis, Roche, Teva; Unterstützung für die Teilnahme an Tagungen und/oder Reisen von Actelion-Janssen, Almirall, Bayer, Biogen, Celgene, Sanofi-Genzyme, Merck, Novartis, Roche, Teva. Teilnahme an einem Data Safety Monitoring Board oder Advisory Board für Sanofi-Genzyme, Biogen, Merck; Leitungs- oder Treuhandfunktion in einem anderen Gremium, einer Gesellschaft, einem Komitee oder einer Interessenvertretung, bezahlt oder unbezahlt als Vorstandsmitglied der Schweizerischen MS-Gesellschaft, Mitglied des medizinisch-wissenschaftlichen Beirats der Schweizerischen MS-Gesellschaft, Mitglied des ärztlichen Beirats der Deutschen MS-Gesellschaft. Erhalt von Geräten, Materialien, Medikamenten, medizinischen Schriften, Geschenken oder anderen Dienstleistungen von Sanofi Genzyme, Biogen, Merck. Sonstige finanzielle oder nicht-finanzielle Interessen z.B. European Journal of Neurology Associate Editor, Clin Transl Neurosci Editorial Board, J Int Med Res Editorial Board.

ACz: Erhielt Beratungshonorare von Biogen, Celgene, Novartis, Roche, Almirall, Merck, TEVA; Zahlungen oder Honorare für Vorträge, Präsentationen, Rednerbüros, Manuskriptschreiben oder Bildungsveranstaltungen von Biogen, Celgene, Novartis, Roche, Almirall, Merck, TEVA; Zahlungen für Expertenaussagen von Biogen, Celgene, Novartis, Roche, Almirall, Merck, TEVA; Unterstützung für die Teilnahme an Tagungen und/oder Reisen von Biogen, Celgene, Novartis, Roche, Almirall, Merck, TEVA. Teilnahme an einem Data Safety Monitoring Board oder Advisory Board für Biogen, Celgene, Novartis, Roche, Almirall, Merck, TEVA; Führungs- oder Treuhänderrolle in einem anderen Gremium, einer Gesellschaft, einem Komitee oder einer Interessengruppe, bezahlt oder unbezahlt für Swiss MS Society Advisory Board.

FD: Erhielt Zuschüsse oder Verträge von Biogen, Roche, Merck, Sanofi, Novartis; Beratungshonorare von Alexion, Almirall, Biogen, Celgene, Sanofi, Merck, Novartis, Roche; Zahlungen oder Honorare für Vorträge, Präsentationen, Referentenbüros, Manuskripterstellung oder Bildungsveranstaltungen von Biogen, Celgene, Sanofi, Merck, Novartis, Roche; Unterstützung für die Teilnahme an Tagungen und/oder Reisen von Biogen, Merck, Sanofi.

FDP: Erhielt Zuschüsse oder Verträge von Roche, Novartis, Merck, Biogen, Genzyme-Sanofi; Zahlungen oder Honorare für Vorträge, Präsentationen, Rednerbüros, Manuskriptschreiben oder Bildungsveranstaltungen von Roche, Novartis, Merck, Biogen, Genzyme-Sanofi, BMS/Celgene; Unterstützung für die Teilnahme an Tagungen und/oder Reisen von Roche, Novartis, Merck, Biogen, Genzyme-Sanofi, BMS/Celgene. Teilnahme an einem Data Safety Monitoring Board oder Advisory Board für Biogen, BMS/Celgene, Genzyme-Sanofi.

RDP: Erklärt, dass kein Interessenkonflikt besteht.

CE: Erhielt Zahlungen oder Honorare für Vorträge, Präsentationen, Rednerbüros, Manuskriptschreiben oder Bildungsveranstaltungen von Novartis, Teva, Biogen, Genzyme, Sanofi, Almirall. Teilnahme an einem Data Safety Monitoring Board oder Advisory Board für Novartis, Biogen, Sanofi.

EF: Erhielt Zahlungen oder Honorare für Vorträge, Präsentationen, Rednerbüros, Manuskriptschreiben oder Bildungsveranstaltungen von Merck, Biogen, Teva; Unterstützung für die Teilnahme an Tagungen und/oder Reisen von Sanofi, Teva, Roche. Teilnahme an einem Data Safety Monitoring Board oder Advisory Board für Celgen, Roche, Merck; Führungs- oder Treuhandfunktion in einem anderen Gremium, einer Gesellschaft, einem Komitee oder einer Interessengruppe, bezahlt oder unbezahlt als ÖGN Vorstand/Präsidentin.

AG: Erhielt Finanzierung und Bereitstellung von Studienmaterialien von Biogen, BMS, Merck, Roche, Schering, Teva; Beratungshonorare von Biogen, BMS, Merck, Roche, Schering, Teva; Zahlungen oder Honorare für Vorträge, Präsentationen, Referentenbüros, Manuskripterstellung oder Bildungsveranstaltungen von Biogen, BMS, Merck, Roche, Schering, Teva.

KG: Erhielt Zahlungen oder Honorare für Vorträge, Präsentationen, Rednerbüros, Manuskriptschreiben oder Fortbildungsveranstaltungen von Genzyme, Roche, Biogen, Novartis; und Unterstützung für die Teilnahme an Tagungen und/oder Reisen, einschließlich DGN 2019, DGN 2020, AAN 2021. Teilnahme an einem Data Safety Monitoring Board oder Advisory Board für Siponimod, Ponesimod, Ozanimod.

CG: Erhielt Honorare für Vorträge, Beratungshonorare und Forschungszuschüsse von Abbvie, Almirall, Biogen Idec, Bayer, Celgene, Genzyme, Merck Serono, Novartis, Teva Pharma, Roche.

NG: Bezahlter Berater, Experte, Adboard-Tätigkeit für Roche; Honorare für Vorträge / Schulungen / Autorenschaften von Biogen; Forschungsprojekte / klin. Studien mit Biogen, Novartis, Roche, Sanofi/Genzyme.

MG: Erhielt Zahlungen oder Honorare für Vorträge, Präsentationen, Rednerbüros, Manuskriptschreiben oder Bildungsveranstaltungen von Almirall, Biogen, Celgene, Genzyme, Merck, Novartis, Roche, Sanofi Aventis und TEVA ratiopharm; Unterstützung für die Teilnahme an Tagungen von Bayer, Biogen, Celgene, Genzyme, Merck, Novartis, Roche, Sanofi Aventis und TEVA ratiopharm. Teilnahme an einem Data Safety Monitoring Board oder Advisory Board für Almirall, Biogen, Celgene, Genzyme, MedDay, Merck, Novartis, Roche, Sanofi Aventis, Shire und TEVA ratiopharm; Führungs- oder Treuhänderfunktion in einem anderen Gremium, einer Gesellschaft, einem Komitee oder einer Interessengruppe, bezahlt oder unbezahlt für Austrian MS Treatment Registry.

AH: Erhielt Zahlungen oder Honorare für Vorträge, Präsentationen, Rednerbüros, Manuskriptschreiben oder Bildungsveranstaltungen von Biogen, Novartis, Celgene, Merck Serono.

HPH: Erhielt Beratungshonorare von Bayer, Biogen, Celgene BMS, MedDay, Merck, Novartis, Roche, TG Therapeutics; Zahlungen oder Honorare für Vorträge, Präsentationen, Rednerbüros, Manuskriptschreiben oder Bildungsveranstaltungen von Novartis, Roche. Teilnahme an einem Data Safety Monitoring Board oder Advisory Board für Novartis, Hoffmann-La Roche.

FH: Erhielt Zuschüsse oder Verträge von Merck; Zahlungen oder Honorare für Vorträge, Präsentationen, Rednerbüros, Manuskripterstellung oder Fortbildungsveranstaltungen von Biogen, Sanofi, Novartis; Teilnahme an einem Data Safety Monitoring Board oder Advisory Board für Sanofi, Biogen.

OH: Erhielt Zuschüsse oder Verträge von Biogen, Novartis, Sanofi; Beratungshonorare von Merck, Novartis, Roche; Zahlungen oder Honorare für Vorträge, Präsentationen, Rednerbüros, Manuskripterstellung oder Bildungsveranstaltungen von Alexion, Biogen, Merck, Novartis , Roche, Sanofi; Unterstützung für die Teilnahme an Tagungen von Bayer, Biogen, Celgene. Teilnahme an einem Data Safety Monitoring Board oder Advisory Board von Biogen, Celgene, Merck, Novartis, Roche, Sanofi; Führungs- oder Treuhänderfunktion in einem anderen Gremium, einer Gesellschaft, einem Ausschuss oder einer Interessengruppe, bezahlt oder unbezahlt als Ärztlicher Beirat, Deutsche Multiple Sklerose Gesellschaft Brandenburg (unbezahlt). Erhalt von Geräten, Materialien, Medikamenten, medizinischen Schriften, Geschenken oder anderen Dienstleistungen von Merck, Novartis.

ZRH: Erklärt, dass kein Interessenkonflikt besteht.

BK: Erhielt Zahlungen oder Honorare für Vorträge, Präsentationen, Rednerbüros, Manuskriptschreiben oder Bildungsveranstaltungen von Biologix, Biogen, Merck, Sanofi, BMS, Novartis. Teilnahme an einem Data Safety Monitoring Board oder Advisory Board für Biogen, Merck, Sanofi, Roche, Janssen, BMS, Novartis.

CK: Erhielt Zuwendungen oder Verträge von der Deutschen Forschungsgemeinschaft (DFG), der Europäischen Kommission, dem Bundesministerium für Bildung und Forschung (BMBF), Merck Serono GmbH, Biogen GmbH, Roche Pharma GmbH; Beratungshonorare von Alexion, Biogen, Bristol Myers-Squibb, Daiichi Sankyo, Merck Serono, Mylan/Viatris, Novartis, Pfizer, Roche, Sanofi-Aventis, Stada, Teva; Zahlungen oder Honorare für Vorträge, Präsentationen, Rednerbüros, Manuskriptschreiben oder Bildungsveranstaltungen von Alexion, Almirall, Amgen, Amicus, Bayer, Biogen, Biotronik, Boehringer Ingelheim, Bristol Myers-Squibb, Celgene, CSL Behring, Daiichi Sankyo, Desitin, Eisai, Ever Pharma, GE Healthcare, MedDay Pharmaceuticals, Merck Serono, Mylan/Viatris, Novartis, Pfizer, Roche, Sanofi-Genzyme, Siemens, STADA, Stago, Teva; Unterstützung für die Teilnahme an Meetings und/oder Reisen von Biogen, Merck Serono, Teva, Roche, Sanofi-Aventis, Alexion. Teilnahme an einem Data Safety Monitoring Board oder Advisory Board für Alexion, Biogen, Bristol Myers-Squibb, Daiichi Sankyo, Merck Serono, Mylan/Viatris, Novartis, Pfizer, Roche, Sanofi-Aventis, Stada, Teva. Aktien oder Aktienoptionen bei Biontec, Sanofi.

LK: Erhielt Zuwendungen oder Verträge von der Deutschen Forschungsgemeinschaft, dem IZKF Münster, IMF Münster, Biogen, Immunic AG, Novartis, Merck Serono; Zahlungen oder Honorare für Vorträge, Präsentationen, Referentenbüros, Manuskriptschreiben oder Bildungsveranstaltungen von Bayer, Biogen, Genzyme, Grifols, Merck Serono, Novartis, Roche, Santhera, Teva; Unterstützung für die Teilnahme an Meetings und/oder Reisen von Alexion, Bayer, Biogen, Genzyme, Grifols, Janssen, Merck Serono, Novartis, Roche, Santhera, Teva. Teilnahme an einem Data Safety Monitoring Board oder Advisory Board für Alexion, Genzyme, Janssen, Merck Serono, Novartis, Roche.

VIL: Erhielt Zahlungen oder Honorare für Vorträge, Präsentationen, Rednerbüros, Manuskriptschreiben oder Bildungsveranstaltungen von Novartis, Teva, Biogen, Merck; Unterstützung für die Teilnahme an Tagungen und/oder Reisen von Teva, Novartis.

FL: Erhielt Beratungsgebühren von Almirall, Actelion, Biogen-Idec, Celgene-BMS, MedDay, Merck-Serono, Novartis, Octapharm, Pfizer, Roche, Sanofi-Genzyme, Santhera, Schering, Teva-Ratiopharm; Zahlungen oder Honorare für Vorträge, Präsentationen, Rednerbüros, Manuskriptschreiben oder Bildungsveranstaltungen von Almirall, Actelion, Biogen-Idec, Celgene-BMS, MedDay, Merck-Serono, Novartis, Octapharm, Pfizer, Roche, Sanofi-Genzyme, Santhera, Schering, Teva-Ratiopharm; Zahlungen für Expertenaussagen und Unterstützung für die Teilnahme an Sitzungen und/oder Reisen von Almirall, Actelion, Biogen-Idec, Celgene-BMS, MedDay, Merck-Serono, Novartis, Octapharm, Pfizer, Roche, Sanofi-Genzyme, Santhera, Schering, Teva-Ratiopharm.

VL: Erklärt, dass kein Interessenkonflikt besteht.

JDL: Erhielt Finanzierung, Bereitstellung von Studienmaterialien vom Schweizerischen Nationalfonds (31003A_169664) und der Deutschen Forschungsgemeinschaft (SFB-CRC128); Zuschüsse oder Verträge vom Schweizerischen Nationalfonds (31003A_169664) und der Deutschen Forschungsgemeinschaft (SFB-CRC128); Beratungshonorare von Abbvie, Alexion, Argenx, Biogen, Merck, Novartis, Roche, Sanofi; Zahlungen oder Honorare für Vorträge, Präsentationen, Rednerbüros, Manuskripterstellung oder Bildungsveranstaltungen von Abbvie, Alexion, Argenx, Biogen, Merck, Novartis, Roche, Sanofi; Unterstützung für die Teilnahme an Tagungen und/oder Reisen von Merck. Teilnahme an einem Data Safety Monitoring Board oder Advisory Board für Merck, Roche.

AL: Erhielt Beratungshonorare von Roche, Novartis, Biogen, Almirall, Merck, Celgene, Merck; Zahlungen oder Honorare für Vorträge, Präsentationen, Referentenbüros, Manuskriptschreiben oder Bildungsveranstaltungen von Novartis, Biogen, Almirall, Merck; Unterstützung für die Teilnahme an Tagungen und/oder Reisen von Celgene, Roche, Biogen. Geplante, erteilte oder anhängige Patente: Benannt auf einem Patent der Universität Zürich über die Verwendung von autologen peptidgekoppelten Blutzellen zur Behandlung von MS. Leitende oder treuhänderische Funktion in einem anderen Gremium, einer Gesellschaft, einem Komitee oder einer Interessengruppe, bezahlt oder unbezahlt als Mitglied des Beirats der Schweizerischen MS-Gesellschaft. Aktien oder Aktienoptionen als Mitbegründer und Aktionär von Cellerys.

SGM: Erhielt Zahlungen oder Honorare für Vorträge, Präsentationen, Rednerbüros, Manuskriptschreiben oder Bildungsveranstaltungen von Biogen, Novartis, Sanofi, BMS, Merck Serono, Almirall, Alexion, TEVA, Celgene, Roche; Unterstützung für die Teilnahme an Tagungen und/oder Reisen von Biogen, Novartis, Merck. Teilnahme an einem Data Safety Monitoring Board oder Advisory Board für Biogen, Novartis, Merck.

UML: Erhielt Zuschüsse oder Verträge von Almirall, Bayer Health Care, Biogen Idec, Boehringer Ingelheim, Genzyme, Med Day, Merck Serono, Novartis, Pfizer, Roche, Teva, UCB, Eisai, Mylan, Sanofi Genzyme, Shire, Akcea, Alexion, Alnylam, Behring CSL, Kedrion; Zahlungen oder Honorare für Vorträge, Präsentationen, Rednerbüros, Manuskriptschreiben oder Bildungsveranstaltungen von Roche, Novartis, Alexion, Pfizer, Nordd. Chefärzte, Merck Sereno, Biogen, Lilly, RG Digital, Celgene, Advisory Board Fa. Roche, Streamedup NeuroLive.

MP: Erhielt Beratungshonorare von Novartis; Zahlungen oder Honorare für Vorträge, Präsentationen, Rednerbüros, das Verfassen von Manuskripten oder Bildungsveranstaltungen von Novartis; Unterstützung für die Teilnahme an Tagungen und/oder Reisen von Novartis. Teilnahme an einem Data Safety Monitoring Board oder Advisory Board für Roche.

PR: Erhielt Beratungshonorare von Merck, Sanofi, Roche, Teva; Zahlungen oder Honorare für Vorträge, Präsentationen, Rednerbüros, Manuskripterstellung oder Fortbildungsveranstaltungen von Merck, Sanofi, Roche, BMS, Teva, Celgene, Novartis.

SSch: Erhielt Beratungshonorare von Biogen, Merck Serono, Novartis, Roche; Zahlungen oder Honorare für Vorträge, Präsentationen, Rednerbüros, Manuskriptschreiben oder Bildungsveranstaltungen von BayerVital, Biogen, Merck Serono, Novartis, Sanofi, Roche, Teva; Unterstützung für die Teilnahme an Tagungen und/oder Reisen von BayerVital, Biogen, Merck Serono, Novartis, Sanofi, Roche, Teva. Teilnahme an einem Data Safety Monitoring Board oder Advisory Board für Biogen, Merck Serono, Novartis, Roche.

HT: Erhielt Zuwendungen oder Verträge von BMBF, DMSG, DZNE, GBS-Stiftung, Chemische Fabrik Karl Bucher, Biogen, Genzyme-Sanofi, Merck, Novartis, Roche; Beratungshonorare von Labor Gärtner, Reck; Zahlungen oder Honorare für Vorträge, Präsentationen, Rednerbüros, Manuskriptschreiben oder Bildungsveranstaltungen von Alexion, Bayer, Biogen, Celgene, Genzyme-Sanofi, Merck, Novartis, Roche, Teva. Teilnahme an einem Data Safety Monitoring Board oder Advisory Board für Alexion, Bayer, Biogen, Celgene, Genzyme-Sanofi, Merck, Novartis, Roche, Teva; Führungs- oder Treuhänderrolle in einem anderen Gremium, einer Gesellschaft, einem Komitee oder einer Interessengruppe, bezahlt oder unbezahlt mit der DGLN (Vorstand).

FW: Erhielt Zahlungen oder Honorare für Vorträge, Präsentationen, Rednerbüros, Manuskriptschreiben oder Bildungsveranstaltungen von Pfizer, Merk; Unterstützung für die Teilnahme an Tagungen und/oder Reisen von Novartis, Bayer.

MSW: Erklärt, dass kein Interessenkonflikt besteht.

UKZ: Erhielt Zuschüsse oder Verträge vom BMBF, BMWi, EU; Zahlungen oder Honorare für Vorträge, Präsentationen, Rednerbüros, Manuskripterstellung oder Fortbildungsveranstaltungen von Alexion, Almirall, Bayer, Biogen, Celgene, Genzyme, Merck, Novartis, Roche, Teva. Teilnahme an einem Data Safety Monitoring Board oder Advisory Board für Alexion, Biogen, Celgene, Genzyme, Janssen, Merck, Roche.

TZ: Erhielt Zuwendungen oder Verträge von Novartis, Roche, Teva, Celgene BMS, Sanofi, Biogen, Merck, Alexion, Janssen Cilag; Zahlungen oder Honorare für Vorträge, Präsentationen, Rednerbüros, Manuskriptschreiben oder Bildungsveranstaltungen von Novartis, Roche, Teva, Celgene BMS, Sanofi, Biogen, Merck, Alexion. Teilnahme an einem Data Safety Monitoring Board oder Advisory Board von Roche, Celgene BMS, Sanofi, Biogen, Merck, Novartis; Führungs- oder Treuhänderrolle in einem anderen Gremium, einer Gesellschaft, einem Komitee oder einer Interessengruppe, bezahlt oder unbezahlt als DMSG-Mitgliedschaft, Teilnahme Brain Health Initiative, EAN-Mitgliedschaft, DGN-Mitgliedschaft.

FZ: Teilnahme an einem wissenschaftlichen Beirat oder Vortragstätigkeit für Novartis, Roche, Celgene, Janssen, Novartis Pharma, Janssen Pharmaceutica, Sandoz International, F. Hoffmann-La Roche; Forschungsprojekte/Durchführung von klinischen Studien für Precisis, UCB, SK Life Science, Biogen, Grünenthal, Actelion. Beteiligungen/Anteile im Gesundheitsbereich u.a. Patentanmeldung Cytokin-Derivate.
